# Convergence of Domain Architecture, Structure, and Ligand Affinity in Animal and Plant RNA-Binding Proteins

**DOI:** 10.1093/molbev/msx090

**Published:** 2017-02-25

**Authors:** Raquel Dias, Austin Manny, Oralia Kolaczkowski, Bryan Kolaczkowski

**Affiliations:** 1 Department of Biological Sciences, Northern Arizona University, Flagstaff, AZ; 2 Department of Microbiology & Cell Science, Institute of Food and Agricultural Sciences, University of Florida, Gainesville, FL; 3 Genetics Institute, University of Florida, Gainesville, FL

**Keywords:** ancestral sequence reconstruction, double-stranded RNA binding proteins, protein family evolution, molecular functional evolution, RNA interference

## Abstract

Reconstruction of ancestral protein sequences using phylogenetic methods is a powerful technique for directly examining the evolution of molecular function. Although ancestral sequence reconstruction (ASR) is itself very efficient, downstream functional, and structural studies necessary to characterize when and how changes in molecular function occurred are often costly and time-consuming, currently limiting ASR studies to examining a relatively small number of discrete functional shifts. As a result, we have very little direct information about how molecular function evolves across large protein families. Here we develop an approach combining ASR with structure and function prediction to efficiently examine the evolution of ligand affinity across a large family of double-stranded RNA binding proteins (DRBs) spanning animals and plants. We find that the characteristic domain architecture of DRBs—consisting of 2–3 tandem double-stranded RNA binding motifs (dsrms)—arose independently in early animal and plant lineages. The affinity with which individual dsrms bind double-stranded RNA appears to have increased and decreased often across both animal and plant phylogenies, primarily through convergent structural mechanisms involving RNA-contact residues within the β1–β2 loop and a small region of α2. These studies provide some of the first direct information about how protein function evolves across large gene families and suggest that changes in molecular function may occur often and unassociated with major phylogenetic events, such as gene or domain duplications.

## Introduction

Understanding how proteins evolve novel functional repertoires remains an important goal of molecular and evolutionary biology (Whelan and Goldman 2001; King et al. 2003; Orengo and Thornton 2005). Emerging techniques combining ancestral sequence reconstruction (ASR) with laboratory functional assays and structure determination have allowed researchers to meticulously characterize the evolutionary and structural bases for changes in molecular function (Malcolm et al. 1990; Shih et al. 1993; Ugalde et al. 2004; Bridgham et al. 2006, 2009; Zmasek and Godzik 2011; Voordeckers et al. 2012; van Hazel et al. 2013; Ogawa and Shirai 2014; Whitfield et al. 2015; Clifton and Jackson 2016). While these approaches provide unprecedented opportunities to rigorously investigate the molecular-functional evolution of protein families (Shih et al. 1993; Hanson-Smith et al. 2010; Harms and Thornton 2010; Merkl and Sterner 2016), their reliance on detailed experimental methods limits the scale at which ancestral protein resurrection can be applied.

Several mechanisms can contribute to the generation of new protein functions (Chen et al. 2013), including gene duplication, fission, or fusion (Song et al. 1987; Wang et al. 2004), retrotransposition (Cordaux and Batzer 2009), *de novo* gene origination (Cai et al. 2008), lateral transfer (Dunning Hotopp et al. 2007), shifts in a gene’s reading-frame (Ohno 1984) and domain shuffling (Pao and Saier 1995). The importance of gene duplication for generating molecular-functional novelty across protein families is in little doubt (Saha et al. 2006), even if the particular mechanisms by which duplication allows for functional evolution may be multifaceted (Rastogi and Liberles 2005; Bridgham et al. 2008). Aside from gene dosage effects (Veitia et al. 2013) and post-duplication changes in gene regulation (Nguyen Ba et al. 2014), retention of duplicate genes over long periods of time is generally considered to require significant alteration of at least one duplicate protein’s molecular function (Hughes 1994; Zhang 2003). Post-duplication changes in protein function have been observed in many ASR studies (Tirosh and Barkai 2007; Zhang et al. 2009; Kuraku 2013). Although these findings can be taken as evidence that gene duplication may correlate with functional evolution (Taylor and Raes 2004; Conant and Wolfe 2008; Kassahn et al. 2009), less effort has been invested in looking for functional evolution not associated with gene duplications in large protein families (Bridgham et al. 2008; Hobbs et al. 2012; Bridgham et al. 2014). The low throughput of traditional ASR approaches, coupled with an historical focus on gene duplications, means we have very little unbiased information about how molecular function evolves in large protein families, particularly across deep phylogenetic history.

Here we develop an approach that combines large-scale ancestral sequence reconstruction with molecular dynamics and structure-based affinity prediction to characterize the evolution of molecular function across a large family of double-stranded RNA binding proteins (DRBs). DRBs coordinate the first steps of the RNA interference (RNAi) process, working with Dicer to select dsRNA targets and generate RNA fragments for loading onto the RNA-induced silencing complex (Chendrimada et al. 2005; Liu et al. 2006; Kok et al. 2007; Curtin et al. 2008; Cenik et al. 2011; Fukunaga et al. 2012). Vertebrate DRBs have additionally been shown to regulate cellular stress responses through interactions with Protein Kinase R (Daher et al. 2009; Dickerman et al. 2015). DRBs consist of 2–3 double-stranded RNA-binding motifs (dsrms), short functional domains that either bind double-stranded RNAs or facilitate protein-protein interactions (see supplementary fig. S1, Supplementary Material online) (Kurihara et al. 2006; Laraki et al. 2008; Yang et al. 2010; Wilson et al. 2015). Although DRB function has been examined in a handful of model animals and plants, very little is known about DRB evolutionary history or about how the functional diversity of DRB dsrms evolved (Clavel et al. 2016).

## Results and Discussion

### DRB Protein Families Diversified Independently in Animals and Plants

To begin examining the molecular-functional evolution of double-stranded RNA-binding proteins (DRBs), we identified any protein sequence from NCBI’s NR database encoding 2–3 double-stranded RNA-binding motifs (dsrms) and no other annotated functional domains, consistent with the characteristic domain architecture of DRBs from well-studied model organisms (ang, et al. 2010; Wilson et al. 2015). To construct a reliable consensus phylogeny, we aligned full-length DRB sequences and individual functional domains using a variety of approaches, inferred maximum-likelihood phylogenies from each alignment and combined results using both supermatrix and supertree approaches (see Materials and Methods for details).

**Fig. 1:**
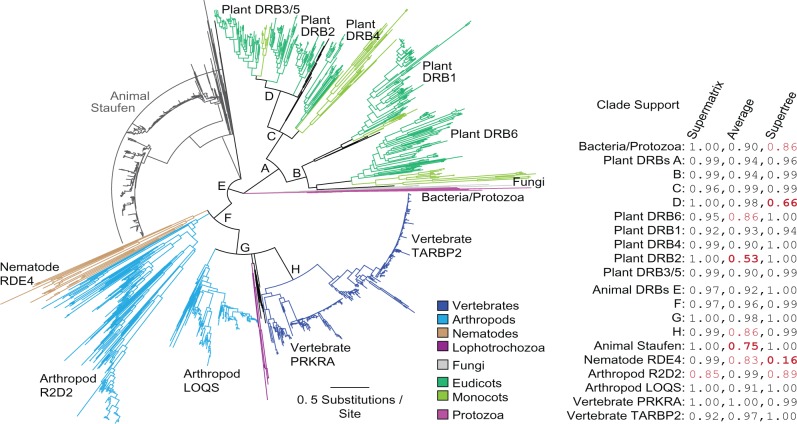
Double-stranded RNA-binding proteins (DRBs) diversified independently in animals and plants. We reconstructed maximum-likelihood phylogenies of all identifiable DRB protein sequences using a variety of alignment strategies and tree reconstruction approaches (see Materials and Methods). We show a consensus tree across all reconstructions. Branch lengths are scaled to the average number of substitutions/site, and major taxonomic groups are indicated by branch color. SH-like aLRT support for major clades is indicated in the table for the supermatrix tree reconstruction, the average support over all individual alignments and the supertree approach (see Materials and Methods); support values <0.9 are red, and values <0.8 are bold. Nodes on the consensus tree are collapsed if they had <0.8 support from all three methods.

A strongly supported consensus phylogeny across all alignment methods and tree-reconstruction approaches suggests that DRB protein families diversified independently early in animal and plant lineages (fig. 1; supplementary file full_trees.nexus.txt contains all trees, and Files DRB_full_idmap.txt and dsrm_full_idmap.txt contain Genbank accession numbers for all sequences, Supplementary Material online). All plant DRBs were monophyletic with >0.94 SH-like aLRT, while animal DRBs grouped with animal Staufen proteins (support >0.92). Within the plant clade, the well-studied DRB1 protein from monocots, dicots and basal vascular plants grouped with a recently characterized DRB6 (support >0.94), but DRB6 has been lost from Brassicaceae (Clavel et al. 2016). Plant DRB4 grouped with an unresolved clade of DRBs from early vascular plants as well as DRB2/3/5 sequences from monocots and dicots (support >0.96), although the DRB2/3/5 clade did not fully resolve in the consensus tree. That sequences from mosses group tightly with DRB1, DRB6 and DRB2/3/5/4 clades suggests that these major gene duplications occurred early in the plant lineage, with later divergence of DRBs 2, 3, and 5, possibly in flowering plants. Given the consensus tree, the timing of DRB4’s origin is unclear; it could have diverged from plant DRB2/3/5 in flowering plants or earlier.

Within the animal clade, DRB sequences from bilateria separated from Staufen proteins and DRB-like proteins from cnidaria with >0.96 SH-like aLRT (fig. 1). While DRBs from arthropods (LOQS) and vertebrates (TARBP2, PRKRA) grouped with lophotrochozoan and invertebrate deuterostome DRBs (support >0.98), the nematode DRB (RDE4) and one of the arthropod DRBs (R2D2) were basal to the main DRB clade (G in fig. 1). This suggests that either the ancestral DRB duplicated early in the bilaterian lineage, with arthropods retaining two DRB genes, nematodes losing one, and the remaining bilateria losing the other, or phylogenetic errors such as long-branch attraction artifactually reshaped the branching pattern of early animal DRB divergence in our analysis.

The grouping of long-branched taxa at the base of a relatively shorter-branched clade is a classic signature of long-branch attraction (Felsenstein 1978; Kuck et al. 2012). However, our previous analysis of Dicer and Argonaute protein families—also participating in RNAi—suggested that these genes also duplicated early in bilateria, with duplicates being lost in non-arthropods (Mukherjee et al. 2013). These results are consistent with a model in which the entire RNAi pathway may have shared an ancient duplication event, followed by lineage-specific losses. Given current results and sequence data, we feel the most appropriate conclusion is to remain agnostic as to the precise pattern of DRB duplications in the animal lineage, although the early divergence of bilaterian DRBs from Staufens appears well-supported, as does a later DRB duplication in the vertebrate lineage (support >0.86; fig. 1).

Although phylogenetic certainty is impossible to completely ensure, and systematic artifacts can generate strongly supported errors in some cases, that the same general tree topology is recovered using different sequence alignments, alignment processing, and tree inference strategies suggests our consensus phylogeny is largely robust to many of the major sources of phylogenetic uncertainty and bias (Zwickl and Hillis 2002; Ogden and Rosenberg 2006). While additional sequence data and major advancements in phylogenetic methods may revise our conclusions in the future, we feel our consensus tree represents a reasonable inference of DRB evolutionary history, given current data, and methodology.

### DRB’s Tandem-dsrm Domain Architecture Arose Independently in Animals and Plants

Animal and plant DRBs have a fairly consistent domain architecture; all well-studied plant DRBs encode two double-stranded RNA-binding motifs (dsrms), whereas animal DRBs encode 2–3 dsrms (Yang et al. 2010; Wilson et al. 2015). No major variations on this 2–3 dsrm domain architecture have been observed, with the recent exception of a possible single-dsrm protein from plants (Clavel et al. 2016). To characterize when and how the DRB domain architecture evolved, we identified all dsrm protein sequences from the NCBI RefSeq database and clustered dsrm proteins by sequence-similarity and phylogenetic analyses to identify those most closely related to dsrms from DRBs (see Materials and Methods, supplementary text S1, tables S1–S3, and fig. S2, Supplementary Materials online). To mitigate potential phylogenetic errors when examining the evolutionary history of short functional domains over long timescales, we used a structural alignment of available dsrm structures and similar folds to align dsrm-related protein sequences for reconstructing the maximum-likelihood domain phylogeny (see Materials and Methods).

**Fig. 2:**
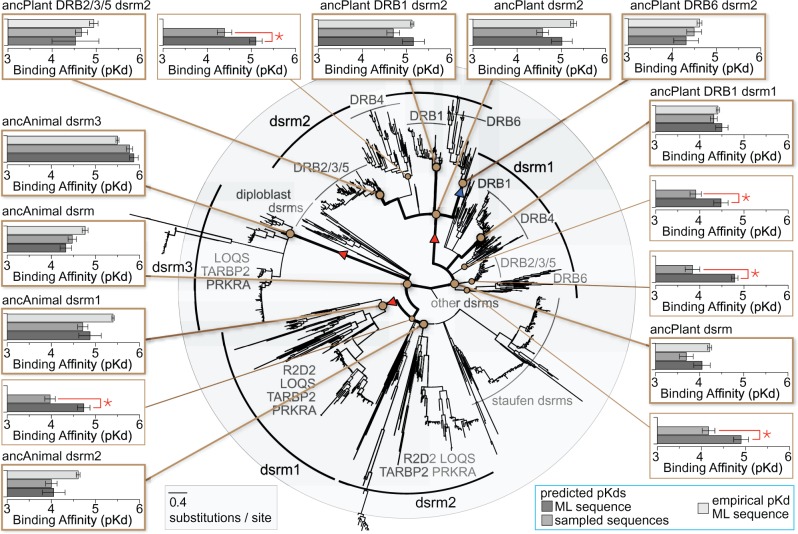
The multiple-dsrm domain architecture of animal and plant DRBs evolved independently, and dsrm–RNA affinities diversified early. We reconstructed the maximum-likelihood domain phylogeny of dsrm functional domains from animal and plant DRB genes, rooted using dsrm domains from other genes and aligned by structure (see Materials and Methods). We plot a consensus tree in which nodes with <0.8 SH-like aLRT are collapsed to polytomies. Branch lengths are scaled to substitutions/site. Ancestral sequences were reconstructed at key nodes on the phylogeny (brown circles), and we inferred the structures of ancestral dsrm protein sequences bound to dsRNA by homology modeling and molecular dynamics; inferred dsrm–RNA complexes were used to predict RNA binding affinities (see Materials and Methods). We plot the predicted dsrm–RNA affinities (p*K*_d_s) of each ancestral sequence, inferred using maximum-likelihood (dark gray bars) or by sampling from the ancestral state posterior distribution (medium gray bars). Light gray bars indicate experimentally determined dsrm–RNA affinities, with standard errors shown (see Materials and Methods for ancestral reconstruction and experimental details). Red triangles indicate significant increases in dsrm–RNA affinities, and blue arrows indicate significant decreases, based on experimentally determined affinity values (*P *<* *0.05). Ancestral nodes for which maximum-likelihood and sampled ancestral sequences had significantly different predicted affinities are indicated by red stars (*P *<* *0.05).

We found that all animal dsrms from DRB proteins were monophyletic (SH-like aLRT = 0.98), all plant dsrms were monophyletic (support = 0.99), and dsrms from animal and plant DRBs were separated from dsrms from other proteins with maximal support (fig. 2, supplementary file full_trees.nexus.txt). Even given the short dsrm sequences, individual dsrm clades were fairly well-supported within animal and plant lineages. The second plant dsrm (dsrm2) was monophyletic with SH-like aLRT = 0.96. Animal dsrm1 and dsrm3 were each monophyletic with support = 0.85 and 0.99, respectively. Aside from dsrm2 from arthropod R2D2, animal dsrm2 domains grouped together with 0.95 support, but the branching order of animal DRB dsrm2s and Staufen dsrms was unresolved. Plant dsrm1 sequences did not form a monophyletic clade with strong support in the consensus phylogeny, but dsrm1 sequences from different plant DRBs did form respective monophyletic groups (support > 0.91). These results are largely consistent with recent phylogenetic analyses of plant DRB and dsrm sequences (Clavel et al. 2016).

Together, our results support a model in which a single ancestral dsrm domain duplicated independently in animal and plant lineages, suggesting that the 2–3 dsrm domain architecture of animal and plant DRBs is a case of convergent evolution. Although we feel the structural alignment is probably more accurate than sequence-based alignments in this case, similar results were obtained using three different sequence alignment strategies, indicating these results are generally robust to alignment ambiguity (supplementary figs. S3–S5, Supplementary Material online). Although support for the monophyletic groupings of dsrm1, dsrm2, and dsrm3 domains was not always high, phylogenetic inferences do not appear to be strongly affected by long-branch attraction or other biases, as major taxonomic groupings tend to follow current species tree estimates. These results generally argue against widespread domain-shuffling or other complex evolutionary scenarios shaping animal or plant DRBs.

Alternatively, the canonical domain architecture could have evolved before the animal–plant split, and partial-gene conversion events or phylogenetic artifacts may be responsible for the apparent respective monophyly of animal and plant dsrms. We did not observe strong evidence for widespread gene conversion among extant DRBs (supplementary table S4, Supplementary Material online). After removing annotated isoforms, we identified 91 pairs of DRB sequences (out of 1793 sequences) that showed significant support for possible gene-conversion events in at least one region (5% of sequences at *P *<* *0.05). Nearly all of these possible gene-conversion events (83) were among closely related mammal TARBP2 sequences, with only three among mammal PRKRA, two among arthropod DRBs, and three among plant DRBs. These results argue against widespread gene conversion affecting the major branching pattern of the dsrm phylogeny, although it may impact the branching pattern within mammalian TARBP2 sequences.

The finding that animal and plant dsrm domains duplicated to produce DRB domain architectures independently in these lineages suggests our initial approach aligning full-length animal and plant DRBs could have introduced potential phylogenetic artifacts (fig. 1). To address this, we inferred separate maximum-likelihood phylogenies of full-length animal and plant DRBs, using respective dsrm outgroup information consistent with the hypothesis that animal and plant DRB domain architectures were independently derived (supplementary fig. S6, Supplementary Material online). These individual animal and plant DRB trees were consistent with the major clades identified in our initial analysis of full-length DRB sequences (see fig. 1, supplementary fig. S6, Supplementary Material online), suggesting our consensus DRB phylogeny is robust.

### High Affinity for RNA Arose Independently in Animal and Plant dsrms

DRB dsrms from model organisms have been observed to play two different functional roles: they bind double-stranded RNA molecules and/or facilitate protein–protein interactions, primarily with Dicer, mammalian PKR or by forming dimers (Kurihara et al. 2006; Laraki et al. 2008; Yang et al. 2010; Wilson et al. 2015). To begin examining how this functional diversity evolved, we reconstructed ancestral protein sequences at early key nodes in the animal and plant dsrm phylogeny, inferred structural complexes with dsRNA by homology modeling, energy-optimized these models by molecular dynamics and predicted dsrm–RNA affinities [p*K*_d_ = −log_10_(*K*_d_)] using a previously developed statistical machine learning approach (see Materials and Methods).

Although maximum-likelihood ancestral sequence reconstruction (ASR) is typically considered robust (Hanson-Smith et al. 2010), some concerns have been raised that choosing the maximum-likelihood state at every position in the ancestral sequence could introduce functional artifacts in some cases, particularly when protein stability is an important component of molecular function (Williams et al. 2006). To address this concern, some researchers have suggested sampling a large number of possible ancestral sequences from the posterior distribution at each site (Pollock and Chang 2007), but to the best of our knowledge, this approach has never been used in practice, due to the cost of experimentally examining the functions of large numbers of ancestral sequences.

As affinity prediction approaches do not suffer from the same efficiency limitations as laboratory analyses, we examined the robustness of affinity estimates to ASR ambiguity by reconstructing multiple “random draws” from each ancestral sequence’s posterior distribution and comparing p*K*_d_ estimates across these posterior-draw sequences to the p*K*_d_ of the maximum-likelihood ancestral sequence, averaged over multiple structural replicates (see Materials and Methods). Nodes for which the predicted RNA affinity of the maximum-likelihood ancestral sequence was not significantly different from the distribution of RNA affinities over random draws were considered robust to ancestral sequence uncertainty; we then expressed the maximum-likelihood protein and measured its affinity for short dsRNA experimentally (see Materials and Methods).

We found that the predicted RNA affinities of 4/5 of the early animal ancestral dsrms were robust to uncertainty in the ancestral sequence reconstruction (at *P *>* *0.05), whereas only 6/10 ancestral plant dsrms were robust to ASR uncertainty (fig. 2). For the cases in which predicted RNA affinities were unaffected by ancestral sequence uncertainty, experimental affinity estimates were generally consistent with maximum-likelihood p*K*_d_ estimates (fig. 2). We observed at most a 3.6-fold difference between experimental and predicted RNA affinity. Only two nodes had >3-fold differences between experimental and predicted affinities (ancAnimal dsrm2 and ancAnimal dsrm1), and only four additional nodes had >2-fold affinity differences (ancAnimal dsrm, ancAnimal dsrm3, ancPlant DRB2/3/5 dsrm2, and ancPlant DRB6 dsrm2).

As figure 2 shows, both animal and plant ancestral dsrms had relatively low affinity for dsRNA (experimentally determined *K*_d_ > 17 μM, *K*_m_ > 16 μM; see supplementary fig. S7, Supplementary Material online) and were statistically indistinguishable from one another (*P *>* *0.34). Ancestral low-affinity for RNA was retained in ancAnimal dsrm2 (*K*_d_ = 24.6 μM, *K*_m_ = 22.9 μM; *P *>* *0.27) and at least one of the ancestral plant dsrm1 lineages (ancPlant DRB4 dsrm1; *K*_d_ = 38.9 μM, *K*_m_ = 38.3 μM; *P *>* *0.29). High affinity for dsRNA (∼10-fold increase) evolved at least once in plants, along the branch leading to ancPlant dsrm2 (*K*_d_ = 5.2 μM, *K*_m_ = 4.2 μM; *P *<* *9.75e^−^^4^) and at least twice in animals, independently along branches leading to ancAnimal dsrm3 (*K*_d_ = 3.2 μM, *K*_m_ = 4.1 μM; *P *<* *4.44e^−^^3^) and ancAnimal dsrm1 (*K*_d_ = 4.0 μM, *K*_m_ = 4.2 μM; *P *<* *1.42e^−^^2^). Finally, ancPlant DRB6 dsrm2 re-evolved low affinity for dsRNA after it diverged from ancPlant dsrm2 (ancPlant DRB6 dsrm2 *K*_d_ = 24.4 μM, *K*_m_ = 24.8 μM; *P *<* *1.17e^−^^2^).

The dsrm structural fold is highly conserved across animals and plants, and structural studies of dsrm–RNA interactions have indicated that dsrms form stabilizing interactions with RNA through two primary interfaces, a loop between β1 and β2, which inserts a canonical histidine into the RNA minor groove, and a cluster of basic residues at the start of α1, which appear to stabilize the RNA backbone (Ryter and Schultz 1998; Yang et al. 2010).

**Fig. 3:**
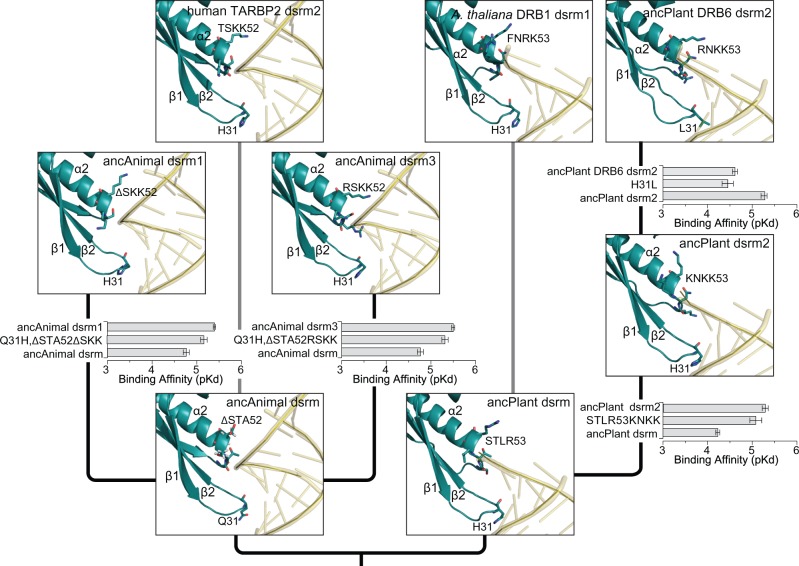
Observed shifts in early animal and plant dsrm–RNA affinities are explained by substitutions in the β1–β2 loop and the α2 region. We reconstructed ancestral animal and plant dsrm protein sequences before and after major shifts in dsrm–RNA affinities (see fig. 2) and predicted the dsrm–RNA structural complex by homology modeling and molecular dynamics (see Materials and Methods). Human TARBP2 dsrm2 and *A. thaliana* DRB1 dsrm1 are shown for comparison. We introduced historical substitutions occurring along the branch spanning each observed functional shift and measured dsrm–RNA affinities using a label-free *in vitro* kinetics assay (see Materials and Methods). We plot the steady-state dsrm–RNA affinity of each protein (p*K*_d_), with longer bars indicating higher affinity. Bars indicate standard errors. Kinetics curves are shown in supplementary figure S9, Supplementary Material online.

Consistent with this model, we found that specific historical substitutions in the β1–β2 loop and the α1 region were responsible for observed changes in dsrm–RNA affinities in animals and plants (fig. 3, supplementary figs. S8 and S9, Supplementary Material online). The ancestral animal dsrm lacked the canonical β1–β2 histidine, had a polar—but not basic—α1 region and bound dsRNA with *K*_d_ = 17.17 μM. Along the branch leading to ancAnimal dsrm3, Q31H, and ΔSTA52RSKK substitutions occurred, which were collectively sufficient to increase dsRNA affinity 4.3-fold in the ancAnimal dsrm background (*P *=* *0.011), making its RNA affinity indistinguishable from that of ancAnimal dsrm3 (*P *=* *0.46). Independent Q31H and ΔSTA52ΔSKK substitutions along the branch leading to ancAnimal dsrm1 were sufficient to increase dsRNA affinity 3-fold (*P *=* *0.013), which was also statistically indistinguishable from the full ancAnimal dsrm1 sequence (*P *=* *0.11). These results suggest that both the ancestral animal dsrm1 and dsrm3 evolved high dsRNA affinity from a low-affinity ancestor through similar structural mechanisms.

Phylogenetic analysis suggests that the evolution of high-affinity dsrm–RNA interactions in animal DRBs occurred through convergent mechanisms, with the H31 substitution arising independently in ancAnimal dsrm1 and dsrm3 as well as along the dsrm2 lineage (see fig. 3, supplementary fig. S8, Supplementary Material online). Although the alternative hypothesis that H31 arose in the common ancestor of animal dsrms is more parsimonious than three independent substitutions, residues flanking H31 are different in ancestral animal dsrm1 and dsrm3 as well as human TARBP2 dsrm2, suggesting that this region can be highly variable (supplementary fig. S8, Supplementary Material online). Ancestral residues at this position were reconstructed with high confidence, arguing against reconstruction uncertainty as a major explanation for this result (supplementary fig. S10, Supplementary Material online). Similarly, the KK54 substitution appears to have occurred independently in animal dsrm1, dsrm3 and dsrm2 lineages, with similar variations in flanking residues and very little uncertainty in ancestral sequences (supplementary figs. S8 and S10, Supplementary Material online). Individual animal dsrm1, dsrm2, and dsrm3 clades were strongly supported phylogenetically using a variety of alignments and inference strategies, arguing against phylogenetic error as the primary explanation for these results (see fig. 2, supplementary figs. S3–S5, Supplementary Material online). Although evolutionary history can never be inferred with absolute certainty, we have not observed any strong evidence for systematic errors in this case.

Although the ancestral plant dsrm had the canonical high-affinity H31 residue (fig. 3, supplementary fig. S8, Supplementary Material online), its STRL53 α2 region was apparently not capable of conferring high dsRNA affinity (*K*_d_ = 59.2 μM). Introducing the derived ancPlant dsrm2 α2 region (KNKK53) into the ancestral plant dsrm background was sufficient to increase dsRNA affinity 9.1-fold (*P *=* *0.021), which was similar to the affinity of ancPlant dsrm2 (*P *=* *0.11). Following the evolution of high RNA affinity in ancPlant dsrm2, an H31L substitution along the branch leading to ancPlant DRB6 dsrm2 re-evolved low RNA affinity (6.6-fold change in *K*_d_; *P *=* *0.031). Together, these results suggest that concerted amino-acid substitutions in the dsrm β1–β2 loop and α1 region were responsible for repeated gains and losses of dsRNA affinity during the early evolution of animal and plant DRBs.

Although most of the critical residues in ancestral β1–β2 loop and α1 regions were reconstructed with high confidence, some critical residues had lower confidence (<0.9 posterior probability), and in some cases, alternative reconstructions with >0.1 probability were identified (supplementary fig. S10, Supplementary Material online). Most alternative reconstructions were within the same biochemical class as the maximum-likelihood residue, and introducing all alternative key residues into the respective maximum-likelihood sequences did not change experimentally determined RNA affinities (*P *>* *0.22). These results suggest that RNA affinity measurements are likely robust to ancestral sequence ambiguity at key residues (see also fig. 2).

Together, our results suggest that the canonical tandem-dsrm architecture of animal and plant DRB proteins was pieced together independently in early animal and plant lineages from an ancestral dsrm that had relatively low affinity for double-stranded RNA. Following early dsrm-domain duplications, independent but similar substitutions in the β1–β2 loop and α1 region of animal (dsrm1, dsrm3) and plant (dsrm2) dsrms produced domains with higher RNA affinity.

Although these results demonstrate quantitative changes in an important component of DRB molecular function, the biological consequences of these changes in dsrm–RNA affinity are difficult to determine. Increases in RNA affinity during early animal dsrm evolution were relatively small (4.3- to 5.4-fold), whereas the change in RNA affinity along the plant dsrm2 branch was more substantial (11.4-fold). Animal and plant DRB proteins coordinate key aspects of the RNA interference process, but how changes in dsrm–RNA affinity might impact RNAi is not known. RNAi plays important roles in animal and plant antiviral immunity by directly targeting viral RNA (Lu et al. 2005; Blevins et al. 2006; Zambon et al. 2006; Segers et al. 2007; Qu et al. 2008; Saleh et al. 2009; Umbach and Cullen 2009), suggesting that even small changes in RNA affinity could impact antiviral RNAi targeting and therefore have a potentially strong effect on organism fitness. RNAi also plays important roles in animal and plant development (Grishok et al. 2001; Ketting et al. 2001; Knight and Bass 2001; Bouche et al. 2006; Kloosterman and Plasterk 2006; Liu et al. 2007; Nag and Jack 2010; Sayed and Abdellatif 2011; Duarte et al. 2013); changes in DRB-RNA affinity could therefore impact developmental timing or progression.

### Dsrm–RNA Affinity Changed Often in Animal and Plant DRB Lineages

To the best of our knowledge, all existing ancestral reconstruction studies have identified particular nodes on the protein family tree to examine based on phylogenetic patterns and/or limited functional analyses of extant proteins. Although productive, existing studies are limited to examining a small number of nodes on the tree and cannot take a comprehensive, unbiased view of how molecular function may have evolved. As a complementary approach, we reconstructed maximum-likelihood ancestral sequences at every node on the dsrm phylogeny, built structural models of each sequence bound to dsRNA, optimized protein–RNA interactions by molecular dynamics and used statistical machine learning to directly infer affinities from the resulting structural complexes (see Materials and Methods). Although computational—rather than experimental—this approach provides a direct assessment of protein–RNA affinity across the entire evolutionary history of DRB dsrm domains, providing a largely unbiased view of how molecular function may have evolved across a large phylogeny.

**Fig. 4:**
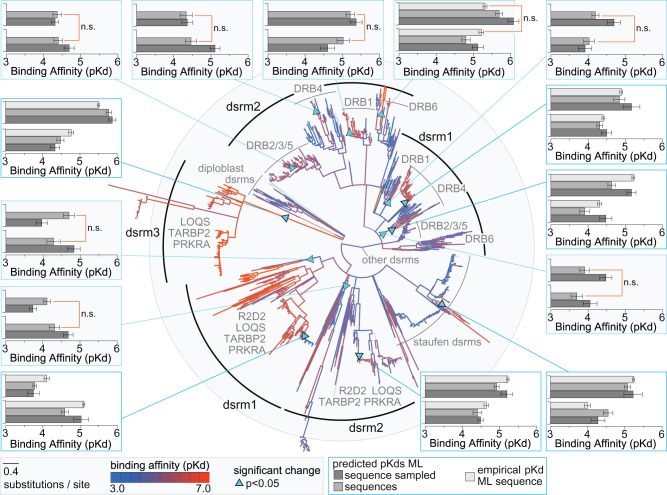
Dsrm–RNA affinities changed often across animal and plant lineages. We inferred the maximum-likelihood phylogeny of dsrm protein sequences using a structure-based alignment (see Materials and Methods). Branch lengths are scaled to substitutions/site, and clades with <0.8 SH-like aLRT are collapsed. Ancestral dsrm sequences were reconstructed at each node on the tree, and dsrm–RNA structural complexes were inferred by homology modeling and molecular dynamics (see Materials and Methods). Dsrm–RNA affinities were predicted by statistical machine learning (see Materials and Methods). We color branches by the average dsrm–RNA binding affinity (p*K*_d_) across multiple replicate models of each ancestral and extant sequence on the phylogeny, with red indicating high-affinity and blue indicating low-affinity. Triangles indicate branches on which there was a significant change in predicted p*K*_d_, as indicated by FDR-corrected independent *t* test. Boxes plot the predicted affinity of the maximum-likelihood ancestral sequence (dark gray), random samples drawn from the ancestral state probability distribution (medium gray) and the experimentally determined affinity (light gray) before (bottom) and after (top) the observed shift. Bars indicate standard errors, and results that were not significant (n.s.) using either sampled sequences or empirical affinity measurements are indicated.

We found that dsrm–RNA affinity appears to have changed significantly and often across animal and plant lineages (fig. 4). The smallest p*K*_d_ estimate was 3.33 (equivalent to *K*_d_ = 467.7 μM), and the largest was 6.53 (*K*_d_ = 0.295 μM), with an average of 4.79 (*K*_d_ = 16.2 μM) and a median of 4.75. Kernel density estimation revealed that the overall distribution of p*K*_d_ estimates was slightly skewed toward marginally smaller values (mode = 4.65), with a noticeable excess of estimates having p*K*_d_ > 5.5 (supplementary fig. S11, Supplementary Material online). We built structural models of dsrm–RNA complexes using human TARBP2 and *Arabidopsis thaliana* DRB1 complexes as templates (see Materials and Methods, supplementary fig. S1, Supplementary Material online). These domains bind RNA in similar conformations (Yang et al. 2010), and p*K*_d_ estimates using each structural template were highly correlated across ancestral and extant dsrm sequences (supplementary fig. S12, Supplementary Material online). Plotting p*K*_d_ estimates from each template on the dsrm phylogeny also revealed similar patterns of high- and low-affinity dsrms (supplementary fig. S13, Supplementary Material online).

Dsrm–RNA affinity prediction used structural information about the dsrm–RNA complex, which we inferred by homology modeling and molecular dynamics (see Materials and Methods). Any errors in ancestral sequence reconstruction that impact protein folding or stability could therefore impact p*K*_d_ prediction. Previous studies have found that ASR errors are associated with high levels of ambiguity in the reconstructed sequence (Hanson-Smith et al. 2010). If p*K*_d_ predictions were strongly affected by error or ambiguity in the ancestral sequence, we would therefore expect a strong correlation between ancestral sequence ambiguity and p*K*_d_ estimates. We found no correlation between p*K*_d_ estimates and the average posterior probability of ancestral states across the phylogeny (Pearson and Spearman correlations <0.02; *P *>* *0.98), suggesting that, overall, ancestral sequence ambiguity did not have a strong effect on p*K*_d_ prediction.

When p*K*_d_ estimates using combined structural templates were plotted on the dsrm phylogeny (fig. 4, supplementary fig. S14, Supplementary Material online), we observed a large number of changes in dsrm–RNA affinity across the tree, with only a few major clades exhibiting stable affinity estimates. The most obvious such grouping was animal dsrm3, which appears to have evolved high affinity for RNA early in its evolutionary history (predicted p*K*_d_ = 5.87 for the ancestral dsrm3 vs. 4.33 for the ancestral animal dsrm; *P *=* *9.39e^−^^5^) and maintained high affinity across all extant and ancestral dsrm3s (mean p*K*_d_ = 5.53, SE = 0.025). Animal dsrm1 also appears to have evolved a relatively stable and high affinity for dsRNA (mean p*K*_d_ = 5.03, SE = 0.035), except in the mammalian TARBP2 lineage, which lost affinity for RNA, according to our analysis (mean p*K*_d_ = 4.00, SE = 0.060). Animal dsrm2’s RNA affinity appeared generally lower than dsrm1 and 3 (mean p*K*_d_ = 4.56, SE = 0.021).

Overall, plant p*K*_d_ predictions were slightly lower than those of animal dsrms (plant mean p*K*_d_ = 4.59, SE = 0.014; animal mean p*K*_d_ = 4.75, SE = 0.018), and we observed fewer large clades with consistently high or low RNA affinities in the plant lineage (supplementary fig. S14, Supplementary Material online). Overall, plant dsrm1 and dsrm2 sequences had similar predicted affinities (dsrm1 mean p*K*_d_ = 4.55, SE = 0.019; dsrm2 mean p*K*_d_ = 4.63, SE = 0.022). Within plant dsrm1 groups, DRB1 had the highest affinity for RNA (mean p*K*_d_ = 4.72, SE = 0.039), and DRB4 had the lowest (mean p*K*_d_ = 4.39, SE = 0.041), but there was only a 2.2-fold variation in average RNA affinities across the major dsrm1 clades (supplementary fig. S14, Supplementary Material online). The major plant dsrm2 clades exhibited a slightly higher variation in RNA affinities (2.9-fold). Similar to results from dsrm1 clades, the dsrm2 domain from DRB1 had the highest affinity for RNA (mean p*K*_d_ = 4.82, SE = 0.039), and DRB4 dsrm2 had the lowest average affinity across the entire clade (mean p*K*_d_ = 4.36, SE = 0.044). There were some smaller plant clades with consistently high RNA affinities (fig. 4). For example, the second dsrm domain of Solanaceae DRB6 had mean p*K*_d_ = 4.93 (SE = 0.195). Aside from Brassicaceae and Rosaceae, the second dsrm domain of eudicot DRB1 also had relatively high affinity for dsRNA (mean p*K*_d_ = 4.92, SE = 0.037).

In order to characterize the rate at which dsrm–RNA affinity evolved across the phylogeny, we treated affinity similar to a quantitative phenotypic trait, applying a Brownian-motion model to infer changes in the rate of affinity evolution across extant and ancestral dsrm domains (Eastman et al. 2011). In general, we expect changes in dsrm–RNA affinity to be roughly correlated with changes in dsrm protein sequence, with significant shifts in the coefficient of proportionality indicating acceleration or deceleration of affinity change, relative to sequence change. We inferred shifts in the coefficient of proportionality using a Bayesian “break point” model across the dsrm phylogeny (see Materials and Methods).

**Fig. 5:**
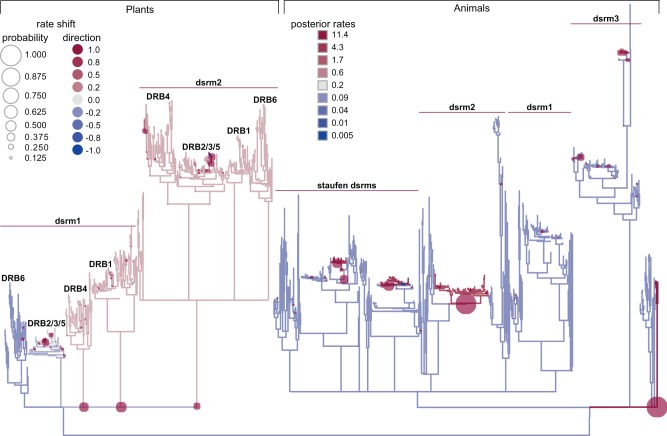
The rate of dsrm–RNA affinity evolution is higher in plants than in animals and exhibits a number of discrete shifts across the dsrm phylogeny. We inferred the evolution of the rate at which dsrm–RNA affinity changes using a Brownian motion “break point” model of affinity evolution fit to predicted dsrm–RNA affinities across extant and ancestral-reconstructed sequences (see Materials and Methods). Branches are scaled to the inferred number of protein substitutions/site and colored by the posterior rate multiplier, averaged over four independent MCMC runs. Red branches indicate faster evolution of dsrm–RNA affinity, with blue branches indicating slower evolution of affinity. Circles on nodes indicate inferred increases (red) or decreases (blue) in the rate multiplier, with the size of the circle indicating the posterior probability of a discrete shift at the specified node. Outgroup branches have been removed. Major taxonomic and gene family lineages are indicated.

We found that—with the exception of early branching dsrm1 sequences from plant DRB6 and DRB2/3/5—plant dsrms had a higher coefficient of proportionality than animal dsrms (fig. 5, supplementary fig. S15, Supplementary Material online), suggesting that changes in dsrm–RNA affinity occurred more often in plants than in animals, relative to dsrm sequence change. Although the inference of strongly supported discrete shifts in the coefficient of proportionality is a known limitation of this type of evolutionary model (Eastman et al. 2011), we did identify a number of discrete increases in the rate of dsrm–RNA affinity change early in the plant lineage (posterior probability >0.35), as well as a spattering of more weakly supported possible changes in more terminal plant lineages (fig. 5). In animal dsrms, we found a strongly supported discrete shift in the rate of dsrm–RNA affinity change in the diploblast lineage (posterior probability =0.92), and another strongly supported shift in the vertebrate TARBP2/PRKRA dsrm2 lineage (posterior probability =0.94; fig. 5). We also observed a number of more weakly supported increases in the rate of dsrm–RNA affinity change across the animal phylogeny (fig. 5). Overall, we observed more support for discrete increases in the rate of dsrm–RNA affinity evolution than decreases. Results were similar when we inferred changes in the rate of dsrm–RNA affinity evolution using the same Brownian-motion model but without considering affinity estimates from ancestral reconstructed sequences, although the absolute rates tended to be marginally lower (supplementary fig. S15, Supplementary Material online).

As a whole, these results suggest that animal and plant dsrm sequences likely evolved under different dynamics. Animal dsrms appear to have differentiated into low- and high-affinity RNA receptors earlier, and affinity was more consistently maintained across larger taxonomic groupings, with an overall reduced rate of affinity change (figs. 4 and 5; supplementary figs. S14 and S15, Supplementary Material online). In contrast, the RNA affinities of plant dsrms appear more evolutionarily labile, with fewer large clades exhibiting high RNA affinity and potentially more variable affinities across major clades.

Prediction of dsrm–RNA affinities across a large phylogeny of ancestral and extant proteins presents an opportunity to directly identify significant shifts in RNA affinities by comparing the p*K*_d_ prediction of each ancestral protein to that of its immediate descendent, thereby identifying particular branches on which dsrm–RNA affinity has changed (see Materials and Methods). This approach may not detect slow changes in dsrm–RNA affinities that occur across multiple branches, and it is unlikely that this approach will have equal power on all branches of the phylogeny. Nonetheless, this simple approach does provide a means for identifying strong, abrupt changes in protein-ligand affinities not linked to specific topological events, such as gene- or domain-duplications.

After correcting for multiple tests, we identified 13 branches across the dsrm phylogeny exhibiting significant support for a shift in RNA affinity, using maximum-likelihood ancestral sequence reconstruction (*P *<* *0.05; fig. 4). Many of these observed shifts in predicted dsrm–RNA affinities were not robust to ancestral sequence ambiguity, particularly in the plant lineage (fig. 4). When we reconstructed multiple replicate ancestral sequences from the posterior distribution (see Materials and Methods), only 3/8 of the inferred shifts in plant dsrm–RNA affinity remained statistically significant, whereas 4/6 shifts observed in the animal lineage were robust to ancestral sequence uncertainty (fig. 4). All but one of the dsrm–RNA affinity shifts that were robust to ancestral sequence ambiguity could be experimentally verified (fig. 4).

Sampling ancestral states from the posterior distribution has been suggested as one approach to alleviate potential state frequency biases in maximum-likelihood ancestral reconstruction (Pollock and Chang 2007). However, the incorporation of low-probability ancestral residues is also expected to introduce a larger number of possible errors, which can collectively degrade protein function (Hobbs et al. 2012). We found that p*K*_d_ estimates obtained from sampled ancestral sequences were almost always the same as or less than estimates using maximum-likelihood ancestral sequences, consistent with a larger number of potential errors introduced by sampling (figs. 2 and 4). Some of the significant shifts in dsrm–RNA preference identified using the maximum-likelihood sequences could in fact be real, even if they failed to be confirmed by posterior sampling (fig. 4). However, here we consider only those shifts found to be robust to ancestral sequence ambiguity.

One of the inferred shifts in animal dsrm–RNA affinity—the shift to high affinity along the branch leading to the dsrm3 lineage—was observed in our earlier analysis (fig. 2) and was found to have occurred via a Q31H substitution in the β1–β2 loop and the introduction of a number of basic residues in the α2 region (fig. 3). Of the remaining three shifts in the animal lineage, one occurred in the Staufen dsrms, and two occurred in early mammals: one a 10.0-fold loss of RNA affinity in mammalian TARBP2 dsrm1 (based on experimentally determined affinities, *P *<* *0.012), and the other a 3.79-fold increase in PRKRA dsrm2’s affinity for RNA (*P *<* *0.036).

**Fig. 6:**
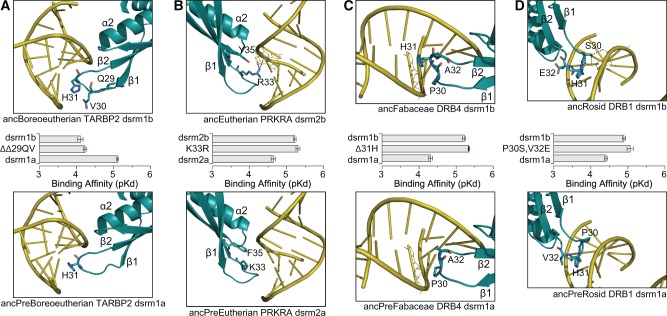
Observed shifts in animal and plant dsrm–RNA affinities are explained by substitutions in the β1–β2 loop and the α2 region. We reconstructed ancestral animal and plant dsrm protein sequences before (bottom) and after (top) major shifts in dsrm–RNA affinities (see fig. 4) and predicted the dsrm–RNA structural complex by homology modeling and molecular dynamics (see Materials and Methods). We introduced historical substitutions occurring along the branch spanning each functional shift and measured dsrm–RNA affinities using an *in vitro* kinetics assay (see Materials and Methods). We plot the steady-state dsrm–RNA affinity of each protein (p*K*_d_), with longer bars indicating higher affinity. Bars indicate standard errors. Kinetics curves are shown in supplementary figure S16, Supplementary Material online.

The loss of RNA affinity in mammalian TARBP2 dsrm1 occurred at the base of the Boreoeutherian lineage. We hypothesized that the insertion of a pair of residues upstream of the RNA-contacting H31 were primarily responsible for the observed loss of RNA affinity by repositioning H31 out of favorable RNA contact (ΔΔ29QV insertion; fig. 6*A*). Indeed, introducing this insertion into the ancestral TARBP2 dsrm1 background reduced RNA affinity nearly 10-fold, which was indistinguishable from that of the derived Boreoeutherian TARBP2 dsrm1 (*P *>* *0.43; fig. 6*A*, supplementary fig. S16*A*, Supplementary Material online). This insertion was strongly supported by ancestral sequence reconstruction (supplementary table S5, Supplementary Material online). The ancestral ΔΔ29 states were reconstructed with posterior probability >0.999, as were the derived QV29 residues.

The second major change in animal dsrm–RNA affinity occurred in the Eutherian mammal PRKRA dsrm2, after the Eutherian mammals diverged from marsupials. In this case, both ancestral and derived PRKRA dsrm2 domains had the canonical H31 RNA-contact residue, although the ancestral mammal PRKRA dsrm2 bound RNA with relatively low affinity (fig. 6*B*). We hypothesized that a single K33R substitution in the dsrm2 β1–β2 loop was responsible for increasing RNA affinity by introducing favorable polar contacts (fig. 6*B*). The ancestral K33 residue was disengaged from the RNA ligand in the structural model, whereas the derived R33 could extend into the RNA’s minor groove to form hydrogen bonds with the RNA base. Consistent with this hypothesis, introducing the K33R substitution into the ancestral mammal dsrm2 background was sufficient to increase dsrm–RNA affinity to that of the derived Eutherian dsrm2 (*P *>* *0.22; fig. 6*B*, supplementary fig. S16*B*, Supplementary Material online). The ancestral K33 residue was reconstructed with posterior probability 0.998, and the derived R33 was reconstructed with posterior probability 1.0, suggesting that ancestral reconstruction ambiguity did not affect this result (supplementary table S5, Supplementary Material online).

We found that similar changes in the β1–β2 loop were responsible for the two observed increases in plant dsrm–RNA affinities (figs. 4 and6*C*–*D*). Both these RNA–affinity shifts occurred in plant dsrm1 lineages, one in Fabaceae DRB4 (fig. 6*C*) and the other in Rosid DRB6 (fig. 6*D*). The ancestral plant DRB4 dsrm1 lacked the canonical H31 RNA-contact residue (reconstructed as Δ31 with posterior probability 0.98; see supplementary table S5, Supplementary Material online) and bound dsRNA with relatively low affinity (experimentally determined p*K*_d_ = 4.31). Introduction of the H31 substitution into this background increased affinity 8.1-fold, which was marginally higher than the derived Fabaceae DRB4 dsrm1 (*P *<* *0.046; fig. 6*C*, supplementary fig. S16*C*, Supplementary Material online). Finally, the ancestral Rosid DRB1 dsrm1 increased RNA affinity 3-fold after Rosids diverged from other plant lineages (from p*K*_d_ = 4.41 to p*K*_d_ = 4.89; fig. 6*D*). This occurred through a pair of substitutions flanking the H31 contact residue, a P30S substitution that introduced favorable dsrm–RNA polar contacts and a V32E substitution (fig. 6*D*). Introducing these substitutions into the ancestral plant DRB1 dsrm1 recapitulated the observed shift in dsrm–RNA affinity along the Rosid lineage (*P *>* *0.37; fig. 6*D*, supplementary fig. S16*D*, Supplementary Material online). As in the animal shifts, all key residues affecting these shifts in plant dsrm–RNA affinity were reconstructed with high confidence, suggesting ancestral sequence ambiguity did not affect these results (supplementary table S5, Supplementary Material online).

Together, these results suggest that convergent evolutionary changes in the β1–β2 region of animal and plant dsrms were responsible for increases and decreases in dsrm–RNA affinities across various animal and plant lineages (figs. 3 and 6; supplementary figs. S9 and S16, Supplementary Material online). These independent changes altered dsrm–RNA affinities through similar structural mechanisms: either by establishing/interfering with a critical H31-RNA contact or by altering dsrm–RNA polar contacts within the β1–β2 loop or α2 region. These findings strongly suggest that the β1–β2 loop is a “hot spot” for “tinkering” with dsrm–RNA affinities across a very broad evolutionary timespan.

We note that not all changes in dsrm–RNA affinities were identified by our phylogeny-wide scan; some of the changes identified during our study of early dsrm diversification were not found (figs. 2 and4). This suggests that the phylogeny-wide scan approach is not a direct replacement for other methods used to identify potential shifts in ancestral molecular function but could be complementary, potentially identifying changes in molecular function not readily predicted by other means. We also note that there are some differences between computationally predicted and experimentally determined p*K*_d_ estimates (figs. 2 and4); this is expected, given that the statistical prediction algorithm was trained across a wide variety of protein–RNA and protein–DNA complexes (Dias and Kolazckowski 2015), and the RNA crystalized with TARBP2 and DCL1 templates is short and may not engage the entire potential RNA-binding region (Ryter and Schultz 1998; Yang et al. 2010). Particularities of the experimental conditions can also have a large effect on affinity measurements (Svec et al. 1980; Reverberi and Reverberi 2007). Nonetheless, the patterns of changes in affinity were generally consistent between computational and experimental approaches, suggesting that computational prediction of protein–RNA affinities is a potentially useful strategy for examining broad-scale changes in molecular function across the evolutionary histories of RNA-binding proteins.

## Conclusions

The continued explosion of “big data” in biology has generated particular challenges that cut across fields; one of which is how best to sort through large, complex data sets to identify specific hypotheses that can be rigorously tested experimentally. Ancestral sequence resurrection studies have historically relied on an ad-hoc assortment of heuristics to identify particular ancestral nodes for functional analysis, including examining gene duplication patterns or patterns of branch lengths, characterizing changes in selection and projecting functional diversity of extant proteins “back in time” along the phylogeny (Malcolm et al. 1990; Shih et al. 1993; Ugalde et al. 2004; Bridgham et al. 2006; Bridgham et al. 2009; Zmasek and Godzik 2011; Voordeckers et al. 2012; van Hazel et al. 2013; Ogawa and Shirai 2014; Whitfield et al. 2015; Clifton and Jackson 2016). While these approaches are useful, they are indirect assessments of the hypothesis under examination, which is when and how molecular function has changed across a protein family’s phylogeny.

Here we have presented a statistical approach for directly examining changes in molecular function across large phylogenies computationally. We have applied this technique to study the evolution of ligand affinity in a family of animal and plant double-stranded RNA binding proteins contributing to RNA interference and demonstrated its capacity to identify shifts in molecular function that were then confirmed experimentally. The scalability of this approach allows researchers to directly examine the effects of ancestral sequence ambiguity and other sources of uncertainty on functional inferences, which is difficult to achieve using low-throughput experiments. We expect that similar computational approaches will help inform future ancestral sequence resurrection studies, ultimately providing a direct and unbiased view of how protein families evolve functional diversity.

Our results demonstrate how individual dsrm functional domains within animal and plant DRB proteins have gained and lost affinity for dsRNA through evolutionary tinkering at two primary dsrm–RNA structural interfaces. However, the implications of these changes in dsrm–RNA affinity for DRB function—or for the functioning of the RNA interference systems they participate in—remain unclear. In addition to binding RNA, DRB dsrms have been shown to directly mediate interactions with Dicers in animals and plants (Kurihara et al. 2006; Wilson et al. 2015), but the extent to which dsrm–RNA and dsrm–protein binding may involve evolutionary “trade-offs” in specialization is not clear. In humans, DRBs appear to interact directly with a short protein-biding domain within the Dicer Helicase (Wilson et al. 2015), potentially altering the structural dynamics and catalytic efficiency of the DRB–Dicer–RNA system, particularly under conditions of high RNA concentrations (Taylor et al. 2013; Fareh et al. 2016). While it is conceivable that changes in dsrm–RNA affinity could impact the functional dynamics of the DRB–Dicer–RNA system, this has not been examined. DRBs have also been shown to help determine specificity of RNA interference pathways in arthropods, although the structural mechanisms are not known (Liu et al. 2006; Zhou et al. 2009; Marques et al. 2010; Hartig and Forstemann 2011). Plant Dicers (aka, “Dicer-like” or “DCL”) lack the protein-binding domain facilitating DRB–Dicer interactions in animals, and appear to interact via dsrm–dsrm contacts (Kurihara et al. 2006), although the structural interface has not been determined. The potential does appear to exist for evolution of DRB function to impact RNA interference through possible effects on Dicer processing of RNA targets. However, further examination of DRB–Dicer–RNA interactions within an explicit evolutionary framework will be required to begin linking specific changes in DRB sequence to potential changes in RNAi processing.

## Materials and Methods

### DRB Sequence Identification, Alignment, and Phylogenetic Analysis

Protein sequences containing at least one double-stranded RNA-binding motif (dsrm, NCBI conserved domain database id CD00048) were identified by rpsblast search of the NR database using an *e*-value cutoff of 0.01 (Marchler-Bauer and Bryant 2004; Marchler-Bauer et al. 2015; Coordinators 2016). Double-stranded RNA-binding proteins (DRBs) were identified as full-length protein sequences containing 2–3 dsrms and no other annotated functional domains with *e*-value <0.01.

Full-length DRB protein sequences were aligned using Clustal Omega v1.2.3 (Sievers et al. 2011), MUSCLE v3.8.31 (Edgar 2004), mafft-einsi v7.215 (Katoh and Standley 2013), and MSAProbs v0.9.7 (Liu et al. 2010) with default parameters. Alignments of only annotated functional domains—with intervening sequence removed—were also produced using the same methods. Alignments were left unprocessed or processed by Gblocks v0.91 to remove potentially ambiguous regions (Talavera and Castresana 2007). We set the minimum number of sequences for a flank position (-b2) equal to 3/5 the total number of sequences in the alignment. The maximum number of contiguous nonconserved positions (-b3) was set to 10. The minimum block length (-b4) was 5, and gap positions were allowed (-b5 = a). Other Gblocks parameters were left at default values.

Initial maximum likelihood phylogenies were constructed from each alignment using FastTree v2.1.7 with default parameters (Price et al. 2010). Initial trees were used as starting trees for full maximum-likelihood reconstruction using RAxML v8.0.24 (Stamatakis 2014), with the best-fit evolutionary model selected from each alignment using AIC in ProtTest v3 (Darriba et al. 2011). Clade support was evaluated by SH-like aLRT scores (Anisimova and Gascuel 2006). Maximum-likelihood phylogenies produced from each alignment were converted to a clade presence–absence matrix using the Super Tree Toolkit v0.1.2 (Hill and Davis 2014), and a supertree was inferred from this matrix using the BINCAT model in RAxML (Nguyen et al. 2012). We also concatenated all individual alignments into a single supermatrix and reconstructed the maximum-likelihood protein family phylogeny using RAxML, with the best-fit evolutionary model selected by AIC (Wheeler et al. 1995). We present a consensus of “supertree” and “supermatrix” results.

### Dsrm Functional Domain Identification, Structural Modeling, and RNA Affinity Prediction

We identified all dsrm functional domains from the RefSeq database (Pruitt et al. 2007) using the approach described in the previous section. Dsrm protein sequences were clustered using MCL v14-137 (Enright et al. 2002). We calculated all-vs.-all blast distances among identified dsrms with an *e*-value cutoff of 0.1. *E*-values were –log_10_-tranformed and capped to ≤200. Node degrees were capped at 280, which was the smallest maximum node degree that maintained a fully connected graph. MCL clustering was performed at various inflation parameters (1.01, 1.05, 1.1, 1.15, 1.2, 1.4, 1.6, 1.8, 2.0, and 3.0) after pre-inflating the graph (-pi 3) to improve contrast between high and low edge weights. Annotated DRBs from *H. sapiens*, *D. melanogaster*, and *A. thaliana* genomes were mapped to clusters, and we selected the optimal MCL clustering as that which maximized the number of annotated DRBs per cluster. All sequences within any cluster containing at least one annotated DRB were considered potential closely related DRB homologs.

Dsrm sequences closely related to those from DRBs were also identified phylogenetically. All dsrm protein sequences were aligned using the methods described above, and maximum-likelihood phylogenies were inferred from each dsrm alignment. Any dsrm sequences grouping with annotated DRBs from *H. sapiens*, *D. melanogaster* and *A. thaliana* with SH-like aLRT > 0.9 were considered closely related, and we combined closely related dsrms from Markov clustering and phylogenetic analysis.

We identified experimentally determined dsrm structures by sequence search of the RCSB protein data bank (Rose et al. 2013), using dsrms from annotated human, *D. melanogaster* and *A. thaliana* DRBs as queries and an *e*-value cutoff of 0.01. Resulting X-ray and NMR structures were aligned using the cealign algorithm in Pymol v1.8.1. We used the mafft –add parameter to align dsrm protein sequences to the structure-based alignment. We inferred the maximum-likelihood dsrm domain tree from the structure-based alignment, collapsed nodes with <0.8 SH-like aLRT support and reconstructed ancestral dsrm sequences at each node on the phylogeny by maximum-likelihood (Yang et al. 1995). We additionally sampled 20 ancestral dsrm sequences at each node from the posterior distribution of residues reconstructed at each site (Pollock and Chang 2007).

For each ancestral and extant dsrm protein sequence, we used MODELLER v9.14 (Eswar et al. 2008) to infer structural models of the dsrm bound to double-stranded RNA, using human TARBP2 (PDB ID: 3ADL) and *A. thaliana* DRB1 (PDB ID: 3ADI) as templates (Yang et al. 2010). Using each template, we constructed 100 potential structural models and selected the best one using the modeler objective function (molpdf), DOPE and DOPEHR scores (Shen and Sali 2006). Each score was re-scaled to units of standard-deviation across the 100 models, and we selected the best model as that with the best average of re-scaled molpdf, DOPE and DOPEHR scores.

Each initial dsrm–RNA structural model was used as a starting point for a short molecular dynamics simulation using GROMACS v4.6.5 (Pronk et al. 2013). We used the amber99sb-ildn force field and the tip3p water model. Initial dynamics topologies were generated using the GROMACS pdb2gmx algorithm with default parameters. Topologies were relaxed into simulated solvent at pH = 7 using a 50,000-step steepest-descent energy minimization. The system was then brought to 300 K using a 50-ps dynamics simulation under positional restraints, followed by pressure stabilization for an additional 50 ps. Simulations were run using Particle-Mesh Ewald electrostatics with cubic interpolation and grid spacing of 0.12 nm. Van der Waals forces were calculated using a cutoff of 1.0 nm. We used Nose–Hoover temperature coupling, with protein, RNA and solvent systems coupled separately and the period of temperature fluctuations set to 0.1 ps. Pressure coupling was applied using the Parrinello–Rahman approach, with a fluctuation period of 2.0 ps. Nonbonded cutoffs were treated using buffered Verlet lists. We selected five complexes from the last 20 ps of each pressure stabilization simulation for affinity prediction.

Dsrm–RNA affinities were predicted from structural complexes using a statistical machine learning approach (Dias and Kolazckowski 2015). Simulated solvent and ions were excluded from the protein–RNA complex, the binding site was identified, and protein–RNA interactions were decomposed into a vector of atom–atom interaction features likely to correlate with binding affinity, as described in (Dias and Kolazckowski 2015). Affinities [reported as p*K*_d_ = −log(*K*_d_)] were predicted using a support vector regression model previously trained using a large number of protein–RNA and protein–DNA complexes with associated experimental affinity measurements. We report the mean of predicted affinities across the five complexes sampled from each dsrm structural model. Differences in predicted p*K*_d_s were assessed using a two-tailed unpaired *t* test, assuming unequal variances and correcting for multiple tests using an FDR correction (Benjamini and Hochberg 1995). We characterized the impact of ancestral sequence ambiguity on predicted protein–RNA affinities by calculating Pearson and Spearman correlations between p*K*_d_ estimates and the average posterior probability of ancestral states at each node. Significance was evaluated using the Student’s *t*-test.

### Brownian Motion Modeling of dsrm–RNA Affinity Evolution

We modeled the evolution of dsrm–RNA affinity using a Brownian motion process (Felsenstein 1973; Eastman et al. 2011), in which we allowed the rate of affinity evolution to be proportional to the number of substitutions/site along each branch of the phylogeny. The coefficient of proportionality was treated as a free model parameter, and we inferred changes in this parameter’s value using reversible-jump Markov chain Monte Carlo (Eastman, et al. 2011). Proposed changes in the coefficient of rate proportionality (i.e., “rate shifts”) were assumed to be inherited by descendent nodes on the phylogeny, unless subsequent rate shifts were also present in a descendent subtree. Four independent MCMC runs were performed using the full model of Brownian motion including jumps with relaxed rates (type = jump-rbm) for 100,000 generations, sampled every 100 generations, and the first 25% of samples were discarded as burnin. We confirmed that the average standard deviation in rate shift posterior probabilities was <0.01 across independent runs, suggesting that MCMC chains had converged to the stationary distribution (Ronquist et al. 2012). We report posterior probabilities combined from all four independent runs. MCMC analyses were conducted using either extant + ancestral affinity predictions (p*K*_d_s, see above) or only using affinity predictions from extant sequences. Standard errors in affinity predictions were included in all Brownian motion models.

### Experimental Measurement of dsrm–RNA Affinity

We generated blunt-ended GC-rich 28-bp RNA molecules *in vitro* using T7 RNA reverse transcriptase and synthetic dsDNA as template. Complementary purified single-stranded RNAs were annealed to produce double-stranded RNA by combining at 1:1 ratio, heating to 95 °C for 5 min and then cooling to 25 °C. Blunt-ended dsRNA was produced by exposure to alkaline phosphatase. The 3′ end of one RNA strand was biotinylated to facilitate kinetics assays using the Pierce™ 3′ End RNA Biotinylation Kit (Thermo).

Ancestral and extant dsrms were expressed in *E. coli* Rosetta™ 2(DE3)pLysS cells using pET-22b(+) constructs, which were verified by Sanger sequencing. Proteins were purified by His-affinity purification and visualized by SDS-page stained with 1% coomassie. Protein concentrations were measured using a linear-transformed Bradford assay (Zor and Selinger 1996).

We measured dsrm–RNA binding using a label-free *in vitro* kinetics assay at pH = 7 (Abdiche et al. 2008; Frenzel and Willbold 2014). Biotinylated RNA molecules were bound to a series of eight streptavidin probes for 5 min, until saturation was observed. Probes were washed and then exposed to 25 µg/ml biocytin to bind any remaining free streptavidin. Each probe was then exposed to dsrms at increasing concentrations in 1× Kinetics Buffer (ForteBio) for 6 min, followed by dissociation in Kinetics Buffer for an additional 4 min before exposure to the next concentration of dsrm protein (Frenzel and Willbold 2014). Molecular binding at each concentration over time was measured as the change in laser wavelength when reflected through the probe in solution, sampled every 3 ms. Two probes were not exposed to dsrm protein as controls to evaluate system fluctuation across the time of the experiment; measurements from these control probes were averaged and subtracted from each analysis probe.

For each replicate experiment, we estimated the dsrm concentration at which ½-maximal steady-state RNA binding was achieved (*K*_d_) by fitting a one-site binding curve to the steady-state laser wavelengths measured across dsrm concentrations at saturation, using nonlinear regression. We additionally fit 1-site association/dissociation curves to the full time-course data in order to estimate the initial rates of RNA binding across dsrm concentrations and used these rates to calculate the dsrm concentration at which the ½-maximal RNA-binding rate was achieved (*K*_m_). *K*_d_s and *K*_m_s were –log_10_ transformed to facilitate visualization, and standard errors across three experimental replicates were calculated. We calculated the statistical significance of differences between *K*_d_s and *K*ms using the two-tailed unpaired *t* test, assuming unequal variances.

### Data Availability

The structural alignment of dsrm domains and all phylogenetic trees reconstructed in this study are available in supplementary file full_trees.nexus.txt, Supplementary Material online with identifiers mapped to NCBI accessions in supplementary files DRB_full_idmap.txt and dsrm_full_idmap.txt, Supplementary Material online. Ancestral-reconstructed sequences are provided in supplementary file ancestral_dsrms.fasta.txt, Supplementary Material online. Software, statistical models, usage tutorials, and protein–RNA affinity predictions are available online at: https://github.com/Klab-Bioinfo-Tools/GLM-Score (last accessed February 21, 2017). Supplementary text, data tables, figures, and references are available in Supplementary File SI_01.pdf, Supplementary Material online.

## Supplementary Material

Supplementary data are available at *Molecular Biology and Evolution* online.

## Supplementary Material

Supplementary Data
