# Supplementary material for: Convergence of Domain Architecture, Structure, and Ligand Affinity in Animal and Plant RNA-Binding Proteins
Source: Mol Biol Evol. 2017 Feb 25;34(6):1429–44. doi: 10.1093/molbev/msx090 (PMC5435087; doi:10.1093/molbev/msx090)
Supplement: Supplementary Data [file msx090_Supp.zip › SI_01.pdf]

Supplementary Information

Supplementary Text

Text S1. Canonical double-stranded RNA-binding proteins (DRBs) are composed of 2-3 tandem double-stranded RNA-binding motifs (dsrms), with no other annotated functional domains (see Supp. Fig. S1). To characterize how this domain architecture evolved, we identified all known dsrm-containing sequences, aligned individual dsrm domains and reconstructed maximum-likelihood domain trees (see Methods). Given the enormous evolutionary breadth of this analysis and the limited information present in short dsrms, we did not expect this approach to provide an unambiguous global phylogeny; rather, we used this large-scale analysis to determine where to focus subsequent efforts. Particularly, we wanted to assess any evidence that might argue against a simple model in which DRBs evolved by gene duplication and speciation.

We found that all the dsrms from animal and plant DRBs clustered together in the maximum-likelihood phylogeny, regardless of the method used for sequence alignment (see Supp. Fig. S2). In all cases, some sequences not annotated as DRBs grouped with annotated DRB sequences, but dsrms from DRBs and other closely-related proteins formed monophyletic groups that were statistically separable from other dsrms with strong support. After removing redundant protein sequences, we identified 6,569 individual dsrm domains. Of these, 889 grouped with annotated DRB sequences from model organisms with >0.9 SH-like aLRT in at least one of the maximum-likelihood phylogenies. Sequences clustering with annotated DRBs were consistent across alignments, with 861 sequences grouping with DRBs using all three alignments.

Sequence-similarity based Markov clustering also grouped dsrms from annotated animal and plant DRBs together, along with a relatively small number of other sequence groups (Supp. Table S1). Using an inflation parameter of 1.05—which we felt provided the best grouping of annotated DRB sequences—resulted in DRBs from well-annotated model organisms being spread across 3 different Markov clusters, which collectively contained 974 total dsrm sequences. Analysis of the resulting Markov clusters suggests that strong cluster information is present in the dsrm sequence similarity network (Supp. Table S2). For example, clustering using inflation=1.05 captured >96% of the edge weight in the sequence similarity network using <13% of the network area (Supp. Table S2). Calculating the split-join distance between different clusterings also supported the conclusion that dsrm sequence data are strongly clustered; at most 110 split/join operations were required to transform any other clustering into a sub-clustering of the inflation=1.05 network (Supp. Table S3). Across all clusterings, at most 184 operations were required to transform any clustering into a sub-clustering of any other network.

The identification of dsrms from DRBs and other closely-related sequences was very consistent across phylogenetic and Markov clustering approaches, with all 861 sequences grouping with DRBs phylogenetically also clustering with well-annotated DRBs in the Markov analysis. The finding that all identifiable dsrms from DRB sequences are closely related to one another suggests that DRBs did not arise through grafting of dsrms from radically different phylogenetic origins.

Supplementary Tables

Cluster Parameter (I)	No. Sequences in Clusters with DRBs	No. DRBs / Cluster Size
1.01	1601	1/632 10/496 6/282 2/191
**1.05**	**974**	**10/492 5/267 4/215**
1.10	976	10/491 4/247 3/203 1/22 1/13
1.15	965	10/491 4/236 3/205 1/20 1/13
1.20	834	10/491 4/236 3/74 1/20 1/13
1.40	659	4/184 4/151 4/108 1/79 1/44 2/38 1/34 1/11 1/10
1.60	609	4/162 4/151 1/77 3/61 1/44 1/36 2/35 1/22 1/11 1/10
1.80	579	4/150 4/140 1/72 3/61 1/44 1/35 2/35 1/21 1/11 1/10
2.00	573	4/150 4/134 1/72 3/61 1/44 1/35 2/35 1/21 1/11 1/10
3.00	535	4/141 3/104 1/48 2/47 1/44 1/31 1/30 1/27 1/21 1/14 1/10 1/10 1/8

Table S1. Markov clustering of dsrms groups known animal and plant dsrms from DRBs with few other sequences. We used Markov clustering to group dsrms into "domain families" (see Methods). For each value of the cluster "inflation" parameter (I), we show the total number of sequences in any cluster with an annotated animal or plant DRB sequence as well as the total number of annotated DRBs/cluster size. Based on this analysis, we chose the inflation parameter of 1.05 to cluster dsrms (shown in bold).

Cluster Parameter (I)	Efficiency	Mass Fraction	Area Fraction	Number of Clusters	Max Cluster Size	Median Cluster Size	Average Cluster Size
1.01	0.26386	0.97004	0.13118	26	1799	862.6	252.7
**1.05**	**0.28608**	**0.96978**	**0.12558**	**31**	**1795**	**825.8**	**211.9**
1.1	0.29996	0.96682	0.12485	36	1807	821.0	182.5
1.15	0.33885	0.94990	0.10993	46	1664	723.0	142.8
1.2	0.39744	0.91483	0.07167	63	914	471.7	104.3
1.4	0.59991	0.76316	0.02854	147	484	188.4	44.7
1.6	0.62753	0.68863	0.01984	196	349	131.3	33.5
1.8	0.61912	0.64024	0.01694	241	335	112.3	27.3
2.0	0.61555	0.62963	0.01665	270	335	110.4	24.3
3.0	0.56274	0.52325	0.01198	380	337	79.7	17.3

Table S2. Markov clustering inflation parameter (I) affects clustering granularity. For each value of the inflation parameter (I), we characterized the resulting clustering produced for dsrm "domain families." Efficiency measures how much of the total edge weight of the sequence similarity network is captured per area of the resulting clustering. The mass fraction is the total edge weight between nodes in the same cluster divided by the total edge weight in the sequence similarity network. Area fraction is roughly the sum of squares of all cluster sizes divided by the square of the number of sequences. See Methods, (Enright, et al. 2002) for more detailed information. The inflation parameter value of 1.05 was chosen to cluster dsrms (shown in bold).

	1.05	1.1	1.15	1.2	1.4	1.6	1.8	2	3	Merge
**1.01**	345, 100	449, 107	844, 140	1743, 131	3546, 73	4112, 72	4415, 65	4432, 63	4783, 52	4802
**1.05**		146, 52	554, 96	1564, 110	3445, 61	4022, 59	4331, 52	4353, 50	4716, 42	4738
**1.1**			437, 58	1464, 73	3374, 59	3951, 59	4262, 53	4284, 52	4649, 51	4671
**1.15**				1110, 53	3150, 83	3732, 79	4045, 72	4072, 75	4441, 72	4466
**1.2**					2588, 101	3300, 108	3624, 102	3657, 108	4106, 93	4140
**1.4**						1050, 142	1520, 162	1606, 167	2409, 184	2521
**1.6**							591, 102	712, 131	1617, 182	1809
**1.8**								159, 67	1175, 166	1428
**2**									1034, 120	1348

Table S3. Markov clusterings with different inflation parameters (I) were largely overlapping. We plot the pairwise split-join distances between all pairs of dsrm sequence clusterings produced using different values of the inflation parameter (I). The first number in each cell represents the number of split/join operations required to transform the clustering at left into a sub-clustering of the clustering indicated across the top. The second number indicates the number of split/join operations required to transform the clustering at the top into a sub-clustering of the clustering indicated across the left. The final column indicates the number of split/join operations required to transform the left clustering into a sub-clustering of the “merged” clustering, or the maximal clustering that is consistent with all the individual clusterings. See Methods, (Enright, et al. 2002) for more information.

Sequence IDs	Species 1	Species 2	Protein	Simulated P-value	Corrected KA P-value	Begin	End	Length	Polymorphisms	Differences	Total Differences
SEQ0889;SEQ0378	*Apis mellifera*	*Apis dorsata*	TARBP2	0	0	1	212	212	203	0	185
SEQ0239;SEQ0846	*Odobenus rosmarus divergens*	*Cavia porcellus*	TARBP2	0.0154	0.07146	97	130	34	34	0	201
SEQ0846;SEQ0314	*Cavia porcellus*	*Balaenoptera acutorostrata scammoni*	TARBP2	0.0154	0.07146	97	130	34	34	0	201
SEQ0398;SEQ0846	*Ceratotherium simum simum*	*Cavia porcellus*	TARBP2	0.0172	0.08171	97	130	34	34	0	200
SEQ0199;SEQ0491	*Camelus ferus*	*Vicugna pacos*	TARBP2	0	0	1	81	81	72	0	284
SEQ0526;SEQ0618	*Homo sapiens*	*Myotis davidii*	TARBP2	0.0117	0.05189	180	244	65	65	0	119
SEQ0497;SEQ0184	*Arabidopsis thaliana*	*Arabidopsis lyrata subsp. lyrata*	DRB5	0	0	1	56	56	56	0	248
SEQ0449;SEQ0167	*Octodon degus*	*Mustela putorius furo*	TARBP2	0.0034	0.01803	136	212	77	77	0	107
SEQ0167;SEQ0267	*Mustela putorius furo*	*Oryctolagus cuniculus*	TARBP2	0	0	136	254	119	119	0	116
SEQ0876;SEQ0618	*Bos mutus*	*Myotis davidii*	TARBP2	0.0075	0.03537	351	415	65	65	0	121
SEQ0112;SEQ0813	*Gorilla gorilla gorilla*	*Bubalus bubalis*	PRKRA	0.0139	0.06401	328	374	47	47	0	156
SEQ0438;SEQ0016	*Orcinus orca*	*Tursiops truncatus*	TARBP2	0.0208	0.103	1	24	24	24	0	254
SEQ0398;SEQ0314	*Ceratotherium simum simum*	*Balaenoptera acutorostrata scammoni*	TARBP2	0	0	81	322	242	242	0	53
SEQ0162;SEQ0449	*Trichechus manatus latirostris*	*Octodon degus*	TARBP2	0	0.00003	113	270	158	158	0	70
SEQ0194;SEQ0438	*Saimiri boliviensis boliviensis*	*Orcinus orca*	TARBP2	0.0316	0.15818	33	54	22	22	0	265
SEQ0038;SEQ0067	*Odobenus rosmarus divergens*	*Chinchilla lanigera*	TARBP2	0.0001	0.00007	83	108	26	26	0	292
SEQ0194;SEQ0877	*Saimiri boliviensis boliviensis*	*Ceratotherium simum simum*	TARBP2	0.0001	0.0001	1	30	30	30	0	266
SEQ0239;SEQ0314	*Odobenus rosmarus divergens*	*Balaenoptera acutorostrata scammoni*	TARBP2	0.0001	0.00017	132	322	191	191	0	55
SEQ0683;SEQ0830	*Mustela putorius furo*	*Microtus ochrogaster*	TARBP2	0.0001	0.00022	404	433	30	30	0	261

SEQ0162;SEQ0221	*Trichechus manatus latirostris*	*Tarsius syrichta*	TARBP2	0.0001	0.00025	113	270	158	158	0	65
SEQ0438;SEQ0877	*Orcinus orca*	*Ceratotherium simum simum*	TARBP2	0.0001	0.00041	1	30	30	30	0	257
SEQ0438;SEQ0199	*Orcinus orca*	*Camelus ferus*	TARBP2	0.0111	0.05045	37	54	18	18	0	308
SEQ0438;SEQ0150	*Orcinus orca*	*Vicugna pacos*	TARBP2	0.0111	0.05045	37	54	18	18	0	308
SEQ0167;SEQ0221	*Mustela putorius furo*	*Tarsius syrichta*	TARBP2	0.0172	0.08169	136	212	77	77	0	100
SEQ0038;SEQ0369	*Odobenus rosmarus divergens*	*Orycteropus afer afer*	TARBP2	0.0003	0.00126	80	104	25	25	0	280
SEQ0483;SEQ0877	*Gorilla gorilla gorilla*	*Ceratotherium simum simum*	TARBP2	0.0044	0.02273	33	56	24	24	0	266
SEQ0194;SEQ0199	*Saimiri boliviensis boliviensis*	*Camelus ferus*	TARBP2	0.0274	0.13649	37	54	18	18	0	300
SEQ0194;SEQ0150	*Saimiri boliviensis boliviensis*	*Vicugna pacos*	TARBP2	0.0313	0.15395	37	54	18	18	0	299
SEQ0067;SEQ0369	*Chinchilla lanigera*	*Orycteropus afer afer*	TARBP2	0.0004	0.00178	83	104	22	22	0	299
SEQ0483;SEQ0438	*Gorilla gorilla gorilla*	*Orcinus orca*	TARBP2	0.0004	0.00179	5	30	26	26	0	271
SEQ0167;SEQ0221	*Mustela putorius furo*	*Tarsius syrichta*	TARBP2	0.0172	0.08169	136	212	77	77	0	100
SEQ0844;SEQ0911	*Trichechus manatus latirostris*	*Pteropus alecto*	TARBP2	0.0004	0.00196	1	28	28	28	0	258
SEQ0438;SEQ0038	*Orcinus orca*	*Odobenus rosmarus divergens*	TARBP2	0.0014	0.00797	32	54	23	23	0	281
SEQ0877;SEQ0067	*Ceratotherium simum simum*	*Chinchilla lanigera*	TARBP2	0.0007	0.00339	82	105	24	24	0	280
SEQ0186;SEQ0332	*Zea mays*	*Sorghum bicolor*	DRB	0.0011	0.00586	428	446	19	19	0	315
SEQ0457;SEQ0794	*Tupaia chinensis*	*Cricetulus griseus*	TARBP2	0.0012	0.00664	230	360	131	131	0	68
SEQ0844;SEQ0038	*Trichechus manatus latirostris*	*Odobenus rosmarus divergens*	TARBP2	0.0013	0.0071	90	116	27	27	0	255
SEQ0038;SEQ0199	*Odobenus rosmarus divergens*	*Camelus ferus*	TARBP2	0.0131	0.06026	37	56	20	20	0	289
SEQ0438;SEQ0038	*Orcinus orca*	*Odobenus rosmarus divergens*	TARBP2	0.0014	0.00797	32	54	23	23	0	281
SEQ0194;SEQ0016	*Saimiri boliviensis boliviensis*	*Tursiops truncatus*	TARBP2	0.0131	0.05994	33	54	22	22	0	273
SEQ0038;SEQ0150	*Odobenus rosmarus divergens*	*Vicugna pacos*	TARBP2	0.0164	0.07721	37	56	20	20	0	287
SEQ0016;SEQ0150	*Tursiops truncatus*	*Vicugna pacos*	TARBP2	0.0021	0.01114	1	22	22	22	0	286
SEQ0194;SEQ0038	*Saimiri boliviensis boliviensis*	*Odobenus rosmarus divergens*	TARBP2	0.0158	0.07673	33	54	22	22	0	271
SEQ0877;SEQ0150	*Ceratotherium simum simum*	*Vicugna pacos*	TARBP2	0.0024	0.01275	1	22	22	22	0	285
SEQ0016;SEQ0199	*Tursiops truncatus*	*Camelus ferus*	TARBP2	0.0028	0.01457	1	22	22	22	0	284
SEQ0877;SEQ0199	*Ceratotherium simum simum*	*Camelus ferus*	TARBP2	0.003	0.01664	1	22	22	22	0	283
SEQ0449;SEQ0167	*Octodon degus*	*Mustela putorius furo*	TARBP2	0.0034	0.01803	136	212	77	77	0	107
SEQ0701;SEQ0877	*Homo sapiens*	*Ceratotherium simum simum*	TARBP2	0.0044	0.02273	33	56	24	24	0	266
SEQ0483;SEQ0877	*Gorilla gorilla gorilla*	*Ceratotherium simum simum*	TARBP2	0.0044	0.02273	33	56	24	24	0	266
SEQ0194;SEQ0911	*Saimiri boliviensis boliviensis*	*Pteropus alecto*	TARBP2	0.0057	0.02807	1	20	20	20	0	295
SEQ0306;SEQ0890	*Drosophila ananassae*	*Drosophila mojavensis*	R2D2	0.0063	0.03235	148	176	29	29	0	232
SEQ0876;SEQ0618	*Bos mutus*	*Myotis davidii*	TARBP2	0.0075	0.03537	351	415	65	65	0	121
SEQ0438;SEQ0844	*Orcinus orca*	*Trichechus manatus*	TARBP2	0.0079	0.03624	30	51	22	22	0	277

		latirostris									
SEQ0150;SEQ0911	*Vicugna pacos*	*Pteropus alecto*	TARBP2	0.009	0.04129	1	20	20	20	0	292
SEQ0845;SEQ0758	*Cavia porcellus*	*Cavia porcellus*	TARBP2	0.0104	0.04652	1	433	433	424	0	19
SEQ0199;SEQ0911	*Camelus ferus*	*Pteropus alecto*	TARBP2	0.0111	0.04687	1	20	20	20	0	291
SEQ0438;SEQ0199	*Orcinus orca*	*Camelus ferus*	TARBP2	0.0111	0.05045	37	54	18	18	0	308
SEQ0438;SEQ0150	*Orcinus orca*	*Vicugna pacos*	TARBP2	0.0111	0.05045	37	54	18	18	0	308
SEQ0526;SEQ0618	*Homo sapiens*	*Myotis davidii*	TARBP2	0.0117	0.05189	180	244	65	65	0	119
SEQ0844;SEQ0199	*Trichechus manatus latirostris*	*Camelus ferus*	TARBP2	0.0118	0.05317	1	20	20	20	0	290
SEQ0844;SEQ0150	*Trichechus manatus latirostris*	*Vicugna pacos*	TARBP2	0.0118	0.05317	1	20	20	20	0	290
SEQ0194;SEQ0483	*Saimiri boliviensis boliviensis*	*Gorilla gorilla gorilla*	TARBP2	0.0123	0.05341	5	30	26	26	0	246
SEQ0162;SEQ0267	*Trichechus manatus latirostris*	*Oryctolagus cuniculus*	TARBP2	0.0123	0.05724	116	212	97	97	0	82
SEQ0015;SEQ0521	*Tursiops truncatus*	*Erinaceus europaeus*	TARBP2	0.0123	0.05732	1	18	18	18	0	307
SEQ0194;SEQ0016	*Saimiri boliviensis boliviensis*	*Tursiops truncatus*	TARBP2	0.0131	0.05994	33	54	22	22	0	273
SEQ0038;SEQ0199	*Odobenus rosmarus divergens*	*Camelus ferus*	TARBP2	0.0131	0.06026	37	56	20	20	0	289
SEQ0112;SEQ0813	*Gorilla gorilla gorilla*	*Bubalus bubalis*	PRKRA	0.0139	0.06401	328	374	47	47	0	156
SEQ0016;SEQ0038	*Tursiops truncatus*	*Odobenus rosmarus divergens*	TARBP2	0.032	0.1687	32	54	23	23	0	257
SEQ0239;SEQ0846	*Odobenus rosmarus divergens*	*Cavia porcellus*	TARBP2	0.0154	0.07146	97	130	34	34	0	201
SEQ0846;SEQ0314	*Cavia porcellus*	*Balaenoptera acutorostrata scammoni*	TARBP2	0.0154	0.07146	97	130	34	34	0	201
SEQ0194;SEQ0038	*Saimiri boliviensis boliviensis*	*Odobenus rosmarus divergens*	TARBP2	0.0158	0.07673	33	54	22	22	0	271
SEQ0038;SEQ0150	*Odobenus rosmarus divergens*	*Vicugna pacos*	TARBP2	0.0164	0.07721	37	56	20	20	0	287
SEQ0653;SEQ0584	*Ochotona princeps*	*Chrysochloris asiatica*	PRKRA	0.0164	0.07784	133	158	26	26	0	243
SEQ0354;SEQ0867	*Arabidopsis thaliana*	*Arabidopsis lyrata subsp. lyrata*	DRB4	0.0165	0.07803	9	25	17	17	0	314
SEQ0162;SEQ0167	*Trichechus manatus latirostris*	*Mustela putorius furo*	TARBP2	0.0172	0.08169	136	212	77	77	0	100
SEQ0167;SEQ0221	*Mustela putorius furo*	*Tarsius syrichta*	TARBP2	0.0172	0.08169	136	212	77	77	0	100
SEQ0398;SEQ0846	*Ceratotherium simum simum*	*Cavia porcellus*	TARBP2	0.0172	0.08171	97	130	34	34	0	200
SEQ0194;SEQ0491	*Saimiri boliviensis boliviensis*	*Vicugna pacos*	TARBP2	0.0173	0.08672	1	22	22	22	0	270
SEQ0438;SEQ0016	*Orcinus orca*	*Tursiops truncatus*	TARBP2	0.0208	0.103	1	24	24	24	0	254
SEQ0194;SEQ0199	*Saimiri boliviensis boliviensis*	*Camelus ferus*	TARBP2	0.0274	0.13649	37	54	18	18	0	300
SEQ0877;SEQ0369	*Ceratotherium simum simum*	*Orycteropus afer afer*	TARBP2	0.0274	0.14046	83	104	22	22	0	266
SEQ0016;SEQ0067	*Tursiops truncatus*	*Chinchilla lanigera*	TARBP2	0.0312	0.15265	86	104	19	19	0	290
SEQ0194;SEQ0150	*Saimiri boliviensis boliviensis*	*Vicugna pacos*	TARBP2	0.0313	0.15395	37	54	18	18	0	299
SEQ0194;SEQ0438	*Saimiri boliviensis boliviensis*	*Orcinus orca*	TARBP2	0.0316	0.15818	33	54	22	22	0	265

SEQ0016;SEQ0038	*Tursiops truncatus*	*Odobenus rosmarus divergens*	TARBP2	0.032	0.1687	32	54	23	23	0	257
SEQ0701;SEQ0150	*Homo sapiens*	*Vicugna pacos*	TARBP2	0.0342	0.17189	38	56	19	19	0	289
SEQ0844;SEQ0067	*Trichechus manatus latirostris*	*Chinchilla lanigera*	TARBP2	0.0342	0.17189	90	108	19	19	0	289
SEQ0438;SEQ0911	*Orcinus orca*	*Pteropus alecto*	TARBP2	0.0363	0.17925	1	20	20	20	0	280
SEQ0013;SEQ0736	*Pediculus humanus corporis*	*Phoenix dactylifera*	R2D2	0.041	0.203	409	421	13	13	0	349
SEQ0701;SEQ0199	*Homo sapiens*	*Camelus ferus*	TARBP2	0.0414	0.21745	38	56	19	19	0	287
SEQ0483;SEQ0150	*Gorilla gorilla gorilla*	*Vicugna pacos*	TARBP2	0.0414	0.21745	38	56	19	19	0	287

Table S4. Possible gene-conversion events were identified among closely-related mammal TARBP2 proteins. We used GENECONV v1.81 (Sawyer 1989)—with default parameters—to identify potential gene-conversion events among closely-related DRBs. We report all sequence pairs having significant evidence for potential gene conversion in at least one region (simulated p-value < 0.05, based on 10,000 permutations). We also report Karlin-Altschul (KA) p-values for each sequence pair, Bonferroni-corrected for multiple tests. Additional columns display the beginning and ending position of each putative gene-conversion event and its length, the number of polymorphic sites and the number of differences between the two sequences within the gene-conversion region, and the total number of differences between the two coding sequences. We annotate each protein based on its phylogenetic grouping (see Fig. 1, main text).

Protein	ML Residue	Alt. Residue	Protein	ML Residue	Alt. Residue
ancPreBoreoeutherianTARBP2_dsrm1a	Δ29(0.999)	None	ancBoreoeutherianTARBP2_dsrm1b	Q29(1.000)	None
	Δ30(1.000)	None		V30(0.999)	None
ancPreEutherianPRKRA_dsrm2a	K33(0.998)	None	ancEutherianPRKRA_dsrm2b	R33(1.000)	None
ancPreFabaceaeDRB4_dsrm1a	Δ31(0.980)	H(0.020)	ancFabaceaeDRB4_dsrm1b	H31(0.999)	None
ancPreRosidDRB1_dsrm1a	P30(0.980)	S(0.011)	ancRosidDRB1_dsrm1b	S30(0.999)	None
	H31(0.999)	None		H31(0.999)	None
	V32(0.986)	None		E32(0.997)	None

Table S5. Key residues mediating ancestral dsrm-RNA interactions were reconstructed with low ambiguity. For each ancestral sequence examined in Figure 6 (main text), we show the maximum-likelihood (ML) residues reconstructed at key sequence positions, with posterior probabilities in parentheses. Positions for which an alternative reconstruction had >0.01 posterior probability are indicated.

Supplementary Figures and Legends

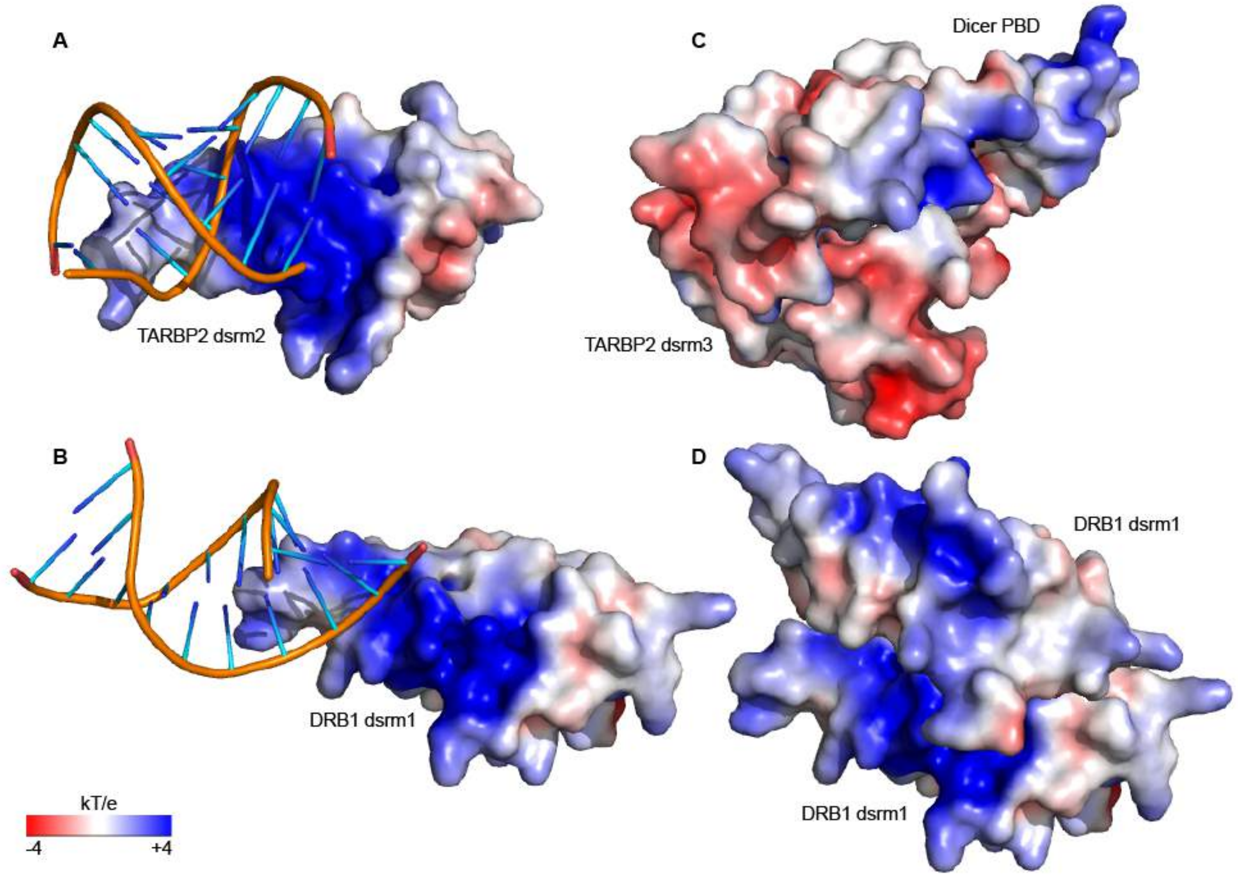

Figure S1. Double-stranded RNA-binding motifs (dsrms) from model animal and plant double-stranded RNA-binding proteins (DRBs) bind RNA and facilitate protein-protein interactions. We plot the electrostatic potential (kT/e) across the molecular surfaces of crystalized dsrm domains (see Methods). Crystal structures of human TARBP2 dsrm2 (**A**, 3ADL) and *Arabidopsis thaliana* DRB1 dsrm1 (**B**, 3ADI) suggest a conserved dsRNA binding interface (Yang, et al. 2010). **C.** We show the crystalized heterodimer of human TARBP2 dsrm3 bound to the protein-binding domain (PBD) of human Dicer (4WYQ) (Wilson, et al. 2015). **D.** Model of a possible dsrm-dsrm dimer inferred from crystalization of *Arabidopsis thaliana* DRB1 dsrm1 domains (3ADI) (Yang, et al. 2010).

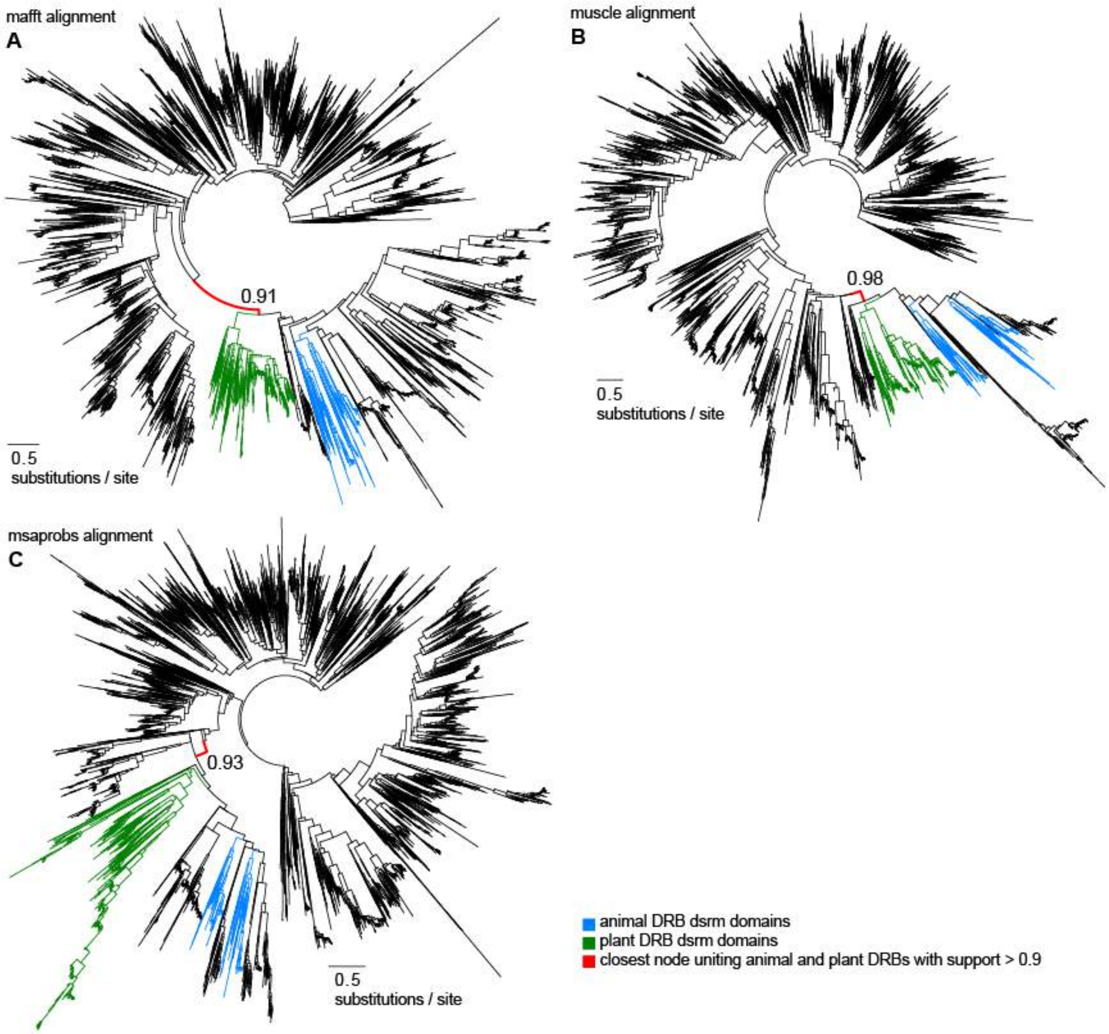

Figure S2. Double-stranded RNA-binding motifs (dsrms) from animal and plant double-stranded RNA-binding proteins (DRBs) cluster phylogenetically with strong support. We inferred the maximum-likelihood phylogeny of all dsrms aligned using mafft (**A**) muscle (**B**) or msaprobs (**C**). See methods for details. We plot the unrooted phylogeny from each analysis. Sequences from animal and plant DRBs are indicated in blue and green, respectively. We indicate the statistical confidence in the closest node uniting all animal and plant DRB sequences with >0.9 SH-like aLRT (highlighted red).

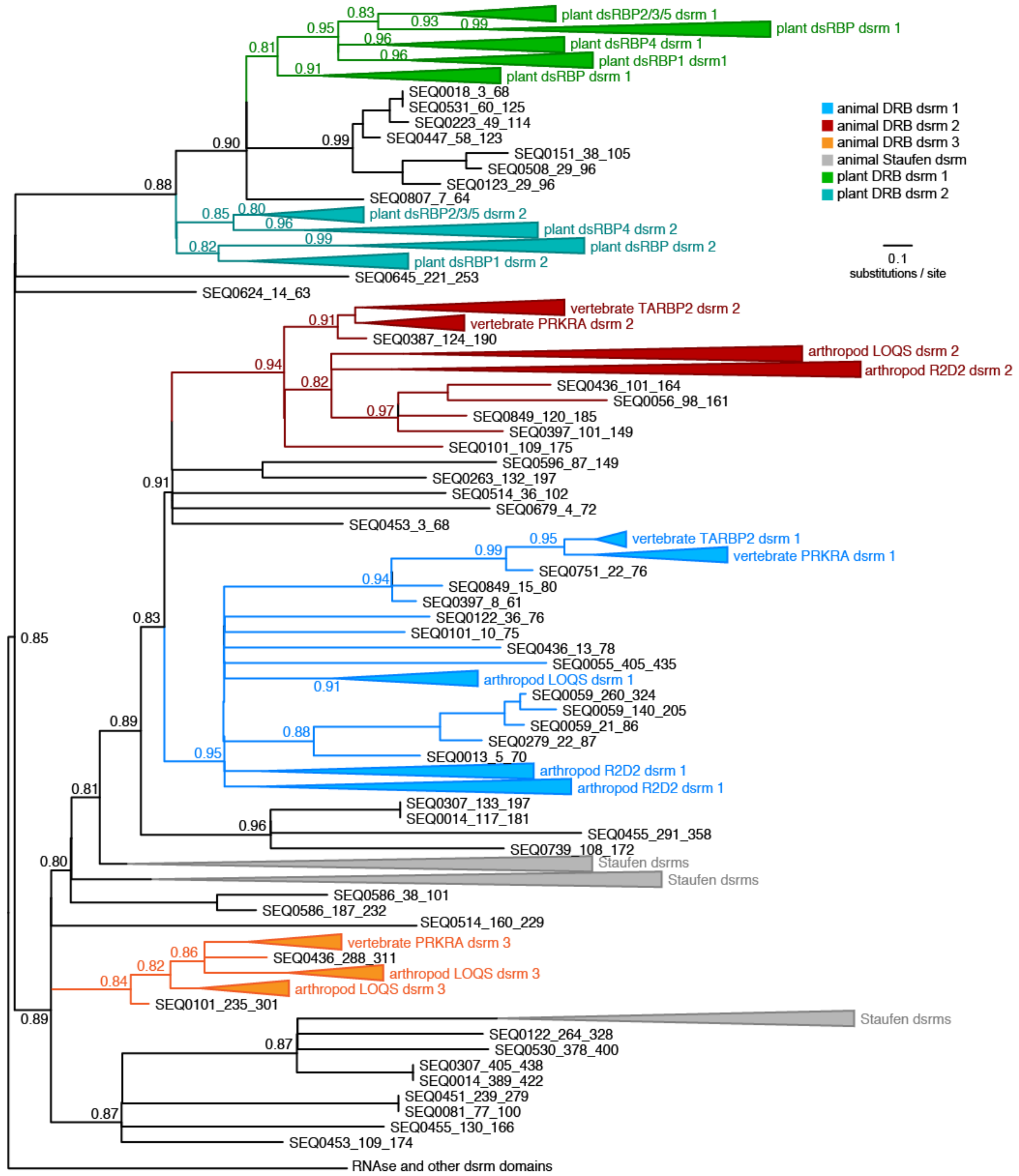

Figure S3. Mafft alignment suggests double-stranded RNA-binding motifs (dsrms) from animal and plant double-stranded RNA-binding proteins (DRBs) are closely related and evolved from a single ancestral dsrm. We used mafft to align all dsrms from animal and plant DRBs—as well as any dsrms that cluster with them by phylogenetic analysis (see Fig. S2) or Markov clustering (see Methods)—and reconstructed the maximum-likelihood phylogeny. We highlight the first (dsrm1), second (dsrm2) and third (dsrm3) dsrm from well-defined DRBs on

the tree, with colors indicating different animal and plant dsrm clades. Clade support is reported as SH-like aLRT scores.

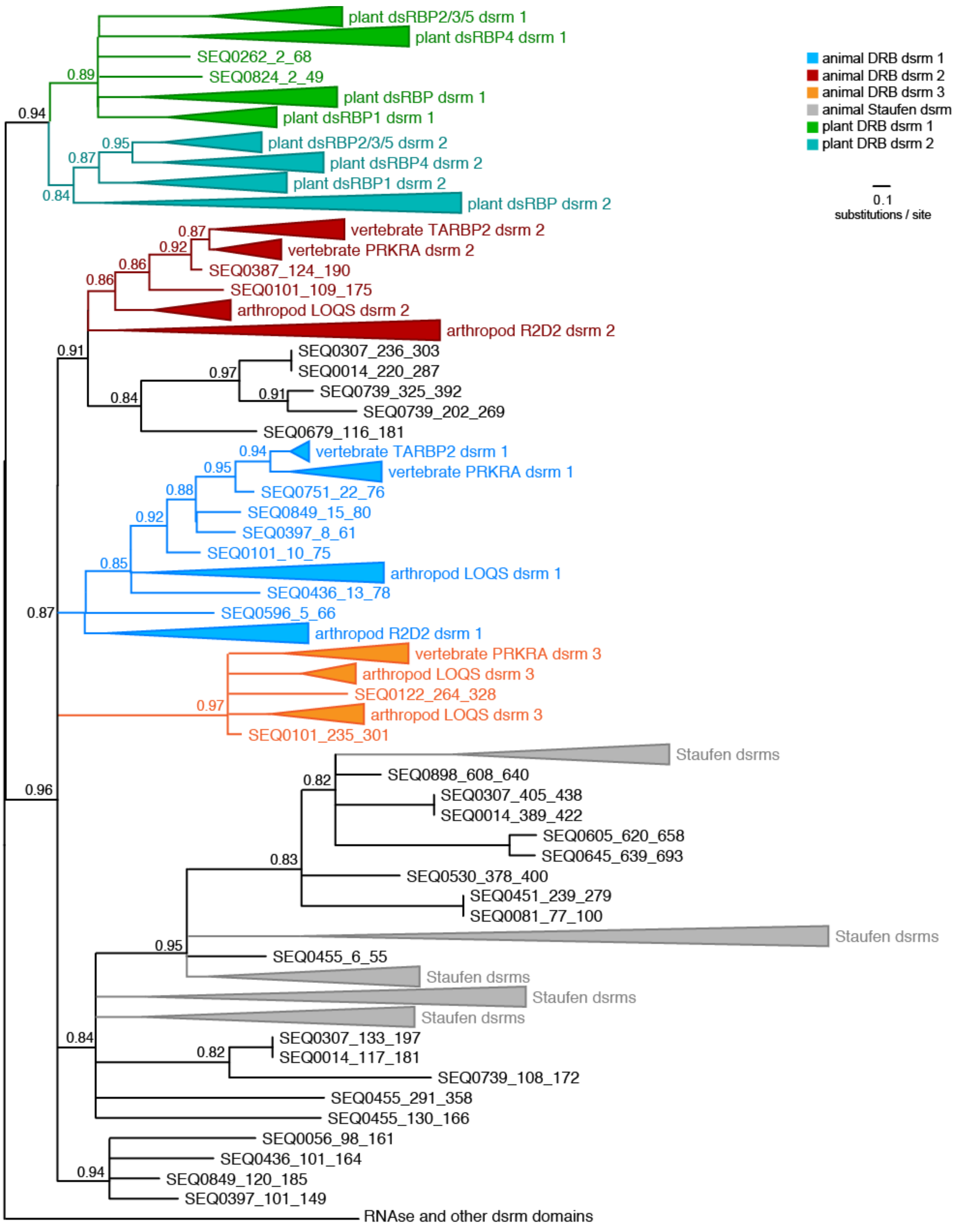

Figure S4. Muscle alignment suggests double-stranded RNA-binding motifs (dsrms) from animal and plant double-stranded RNA-binding proteins (DRBs) are closely related and

evolved from a single ancestral dsrm. We used muscle to align all dsrms from animal and plant DRBs—as well as any dsrms that cluster with them by phylogenetic analysis (see Fig. S2) or Markov clustering (see Methods)—and reconstructed the maximum-likelihood phylogeny. We highlight the first (dsrm1), second (dsrm2) and third (dsrm3) dsrm from well-defined DRBs on the tree, with colors indicating different animal and plant dsrm clades. Clade support is reported as SH-like aLRT scores.

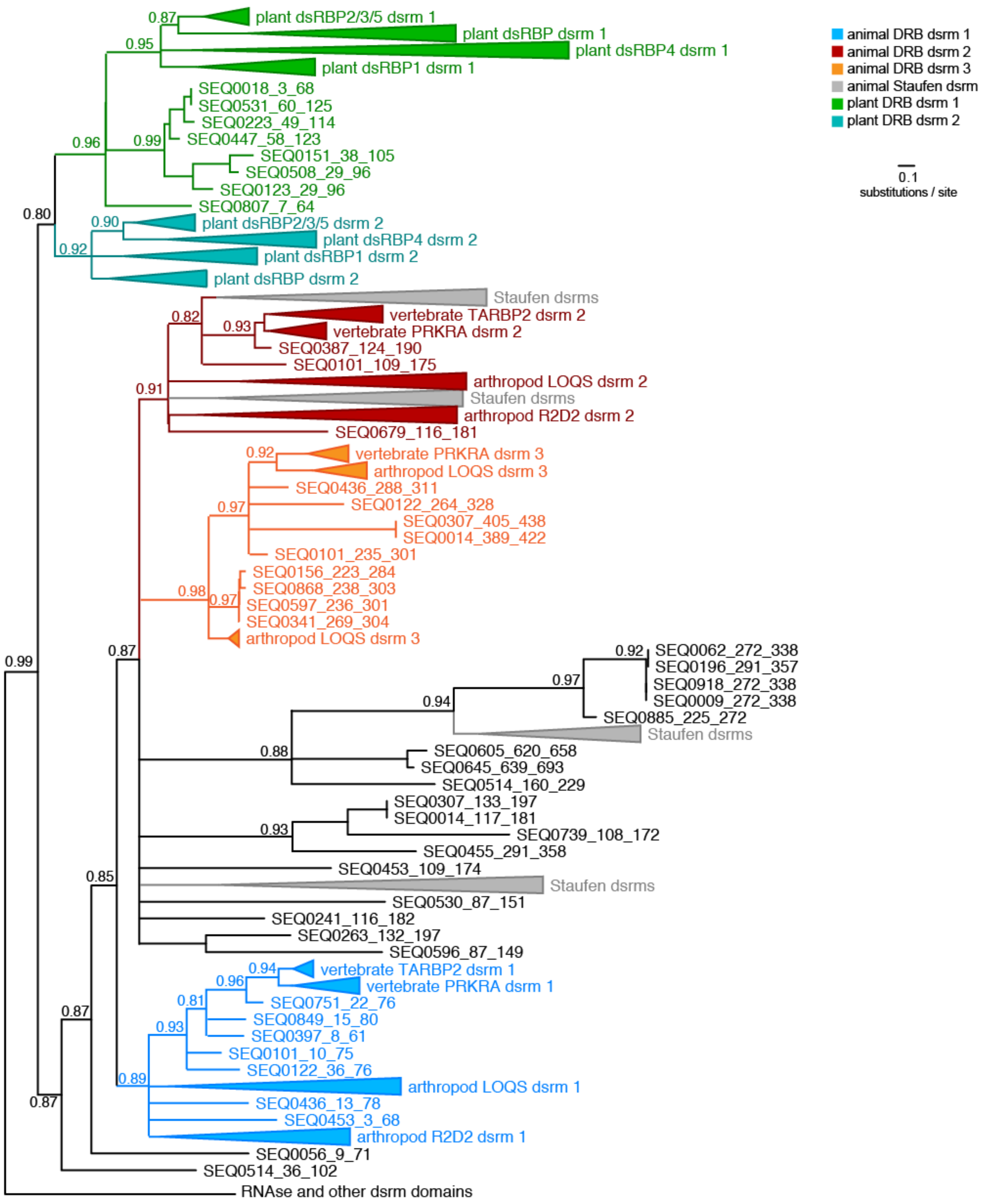

Figure S5. Msaprobs alignment suggests double-stranded RNA-binding motifs (dsrms) from animal and plant double-stranded RNA-binding proteins (DRBs) are closely related and evolved from a single ancestral dsrm. We used msaprobs to align all dsrms from animal and plant DRBs—as well as any dsrms that cluster with them by phylogenetic analysis (see Fig. S2) or Markov clustering (see Methods)—and reconstructed the maximum-likelihood phylogeny. We highlight the first (dsrm1), second (dsrm2) and third (dsrm3) dsrm from well-defined DRBs

on the tree, with colors indicating different animal and plant dsrm clades. Clade support is reported as SH-like aLRT scores.

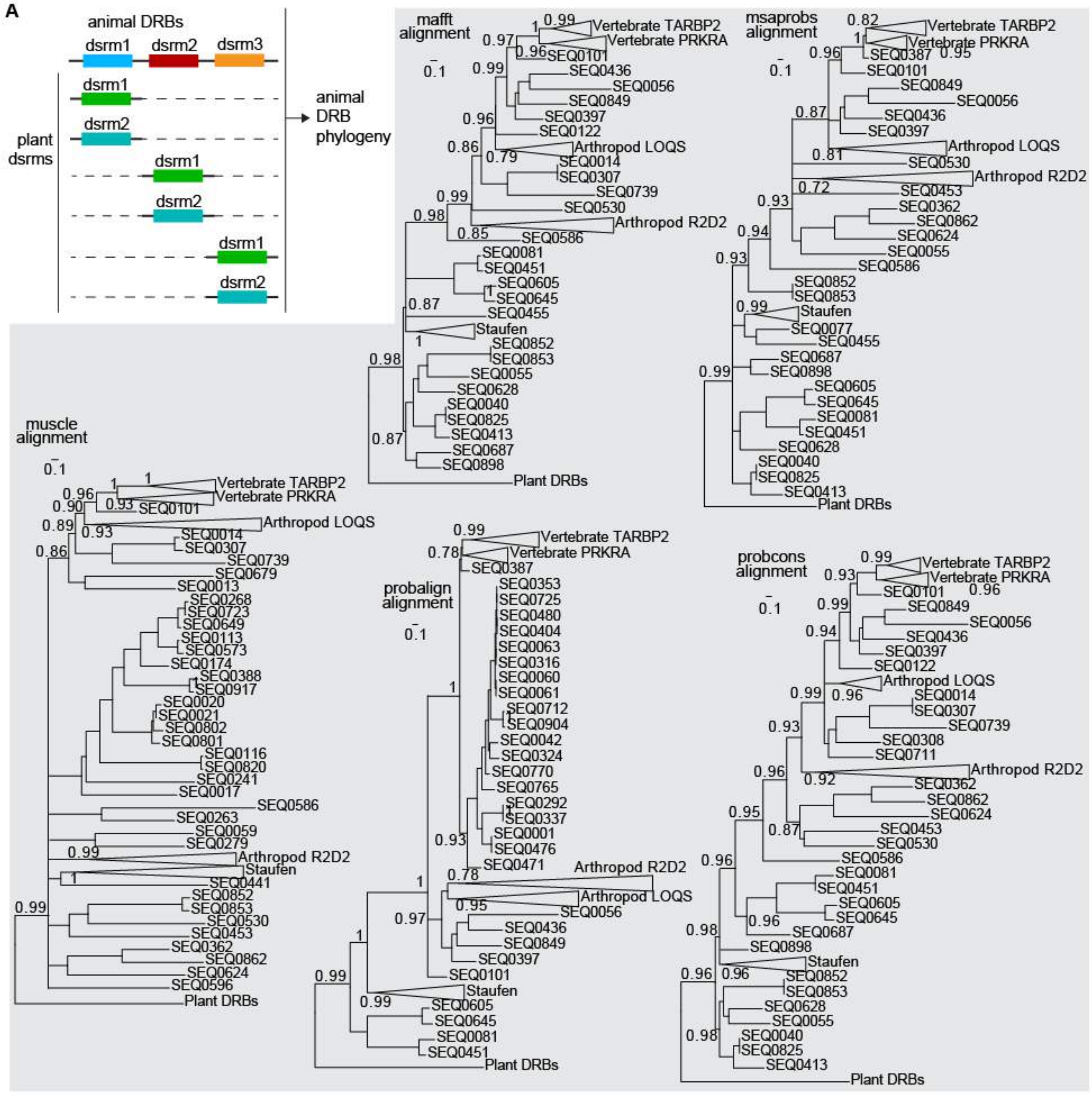

Figure S6, part 1.

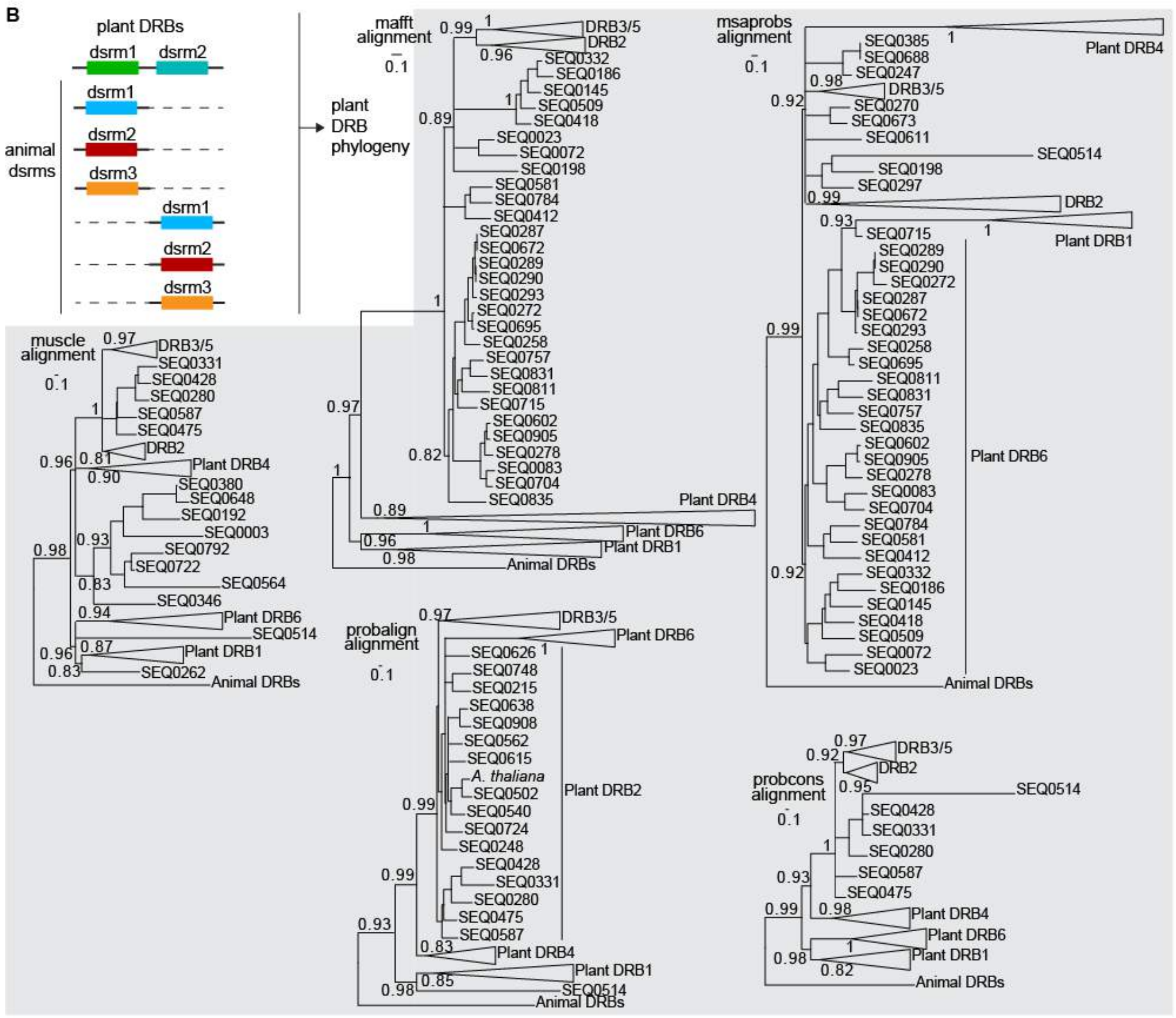

Figure S6. Alignment of full-length animal and plant double-stranded RNA-binding proteins (DRBs) to their respective outgroup dsrms result in phylogenies consistent with the full-length consensus tree (Fig. 1, main text). Phylogenetic analyses suggest that animal and plant DRBs evolved independently from a single ancestral double-stranded RNA-binding motif (dsrm, see Figs. S3-S5). Consistent with this hypothesis, we aligned full-length animal DRBs to individual plant dsrm domains to reconstruct the animal DRB phylogeny, using plant dsrms as an outgroup (**A**). Similarly, we used individual animal dsrms as outgroup sequences to root the full-length plant DRB tree (**B**). We show maximum-likelihood phylogenies, calculated using a variety of alignment methods. Support is indicated by SH-like aLRTs, and branch lengths are scaled to the expected number of substitution/site. Major clades recovered with >0.8 support are collapsed.

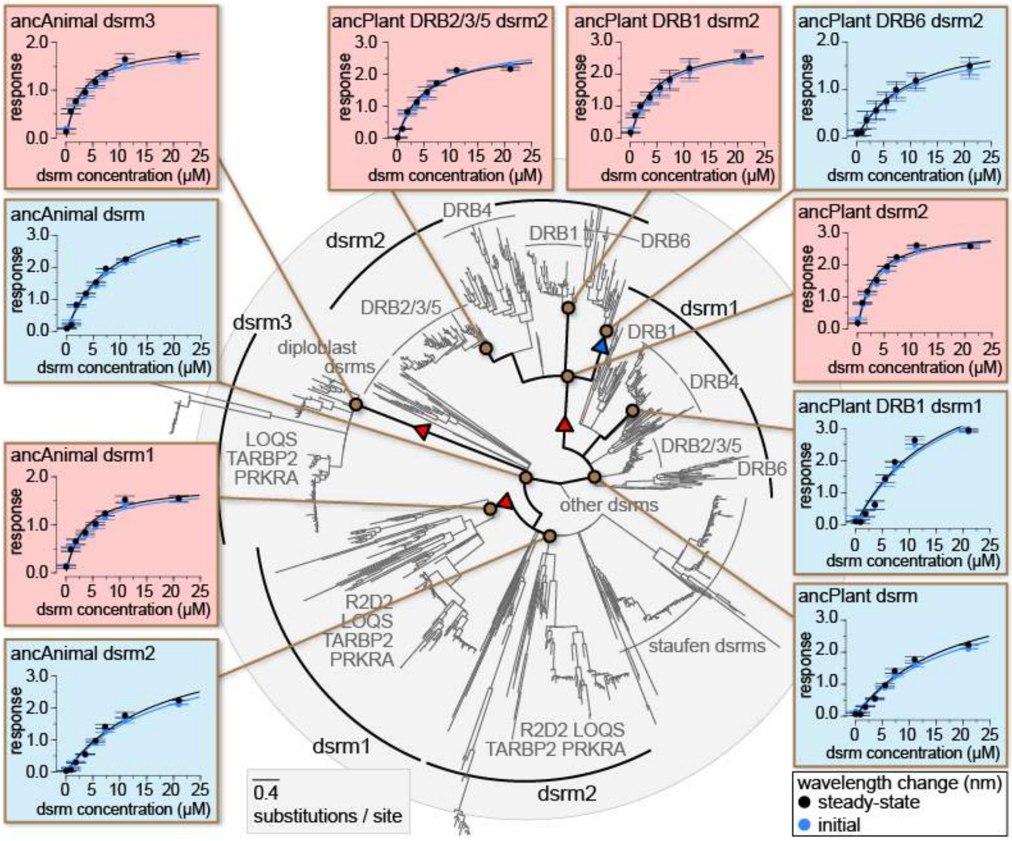

Figure S7. Dsrm-RNA affinities increased independently in early animal and plant lineages. We expressed and purified maximum-likelihood ancestral proteins from key early nodes in the animal and plant dsrm phylogeny (see Fig. 2). We measured steady-state (black) and initial (blue) binding of each ancestral protein to double-stranded RNA using a label-free kinetics assay across increasing dsrm concentrations (see Methods). Lines indicate best-fit single-site binding curves (r^2>0.92), and bars indicate standard errors over three replicates. Red triangles indicate significant increases in dsrm-RNA affinities, and blue arrows indicate significant decreases (p<0.05).

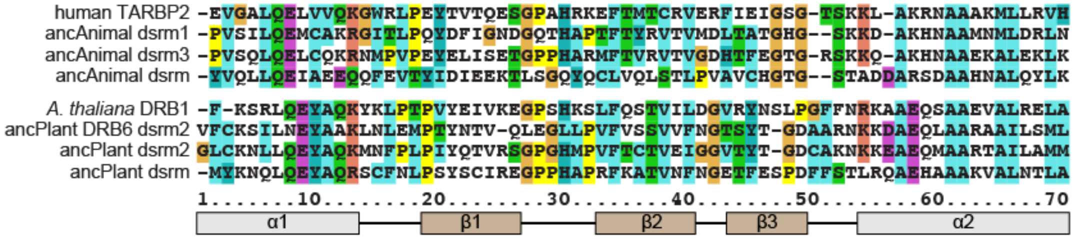

Figure S8. Ancestral animal and plant dsrm domains vary at critical protein-RNA contacts. We plot aligned ancestral animal and plant dsrms examined in Figure 3 (main text), with human TARBP2 dsrm2 and *Arabidopsis thaliana* DRB1 dsrm1 sequences shown for comparison. Secondary structural elements are shown along the bottom of the alignment, with key RNA contacts occurring in the β1-β2 loop and at the beginning of α2 (Yang, et al. 2010).

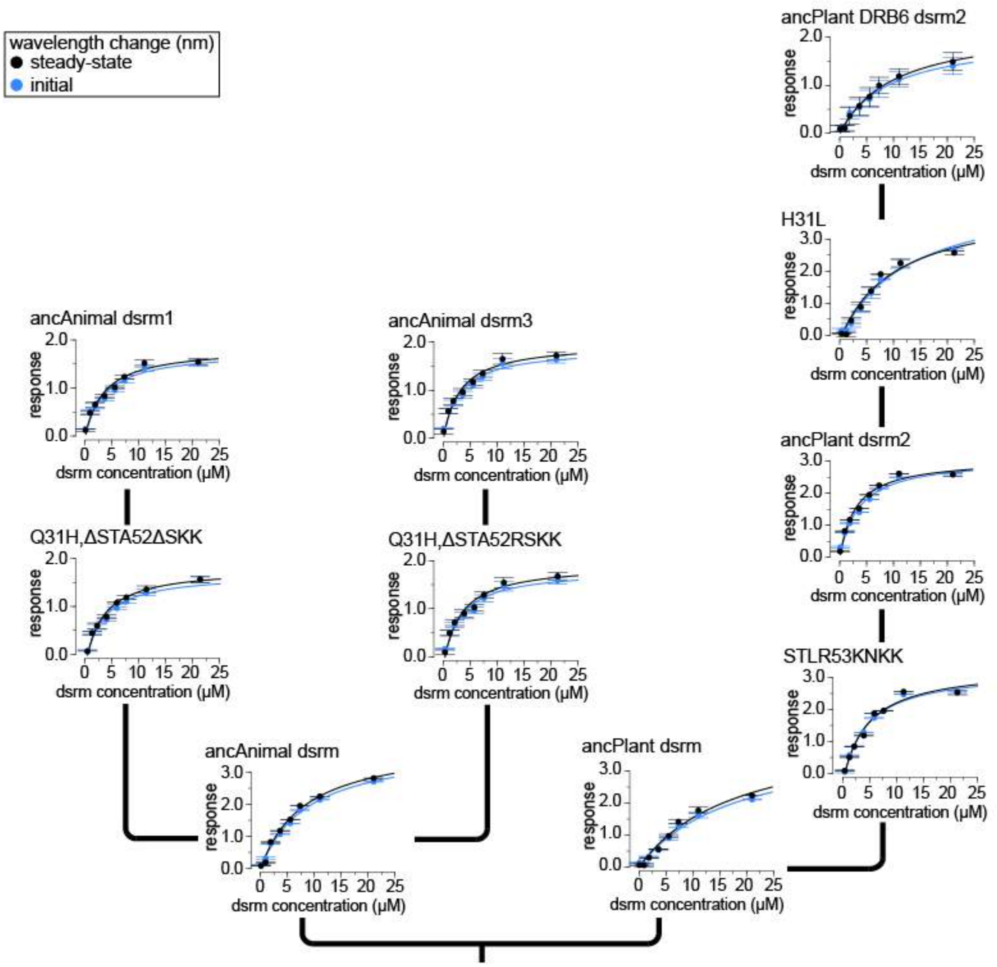

Figure S9. Observed shifts in early animal and plant dsrm-RNA affinities are explained by substitutions in the β1-β2 loop and the α2 region. We measured steady-state (black) and initial (blue) binding of each ancestral protein indicated in Figure 3 (main text) to double-stranded RNA using a label-free kinetics assay across increasing dsrm concentrations (see Methods). Historical substitutions at key RNA-contacting residues were introduced by site-directed mutagenesis. Lines indicate best-fit single-site binding curves (r^2>0.92), and bars indicate standard errors over three replicates.

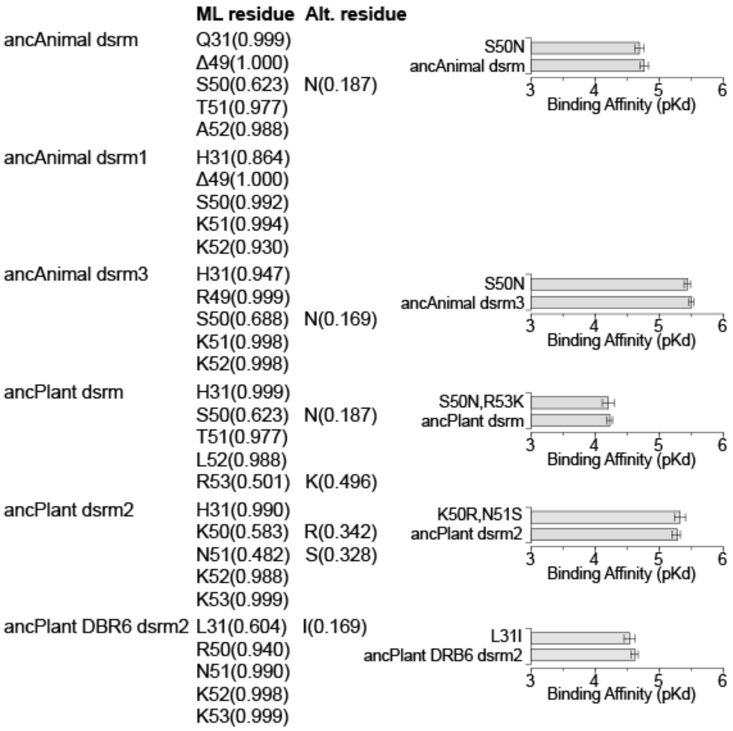

Figure S10. Ancestral reconstruction ambiguity does not affect experimental affinity measurements. For each ancestral sequence examined in Figure 3 (main text), we show the maximum-likelihood residues reconstructed at key sequence positions, with posterior probabilities in parentheses. Positions for which an alternative reconstruction had >0.1 posterior probability are indicated. We reconstructed 'alternative' ancestral sequences by replacing each maximum-likelihood residue with its plausible alternative; bar graphs compare steady-state experimental affinity measurements (pKds) between maximum-likelihood and alternative reconstructions, with longer bars indicating higher affinities. Standard errors over three replicates are indicated (see Methods for details).

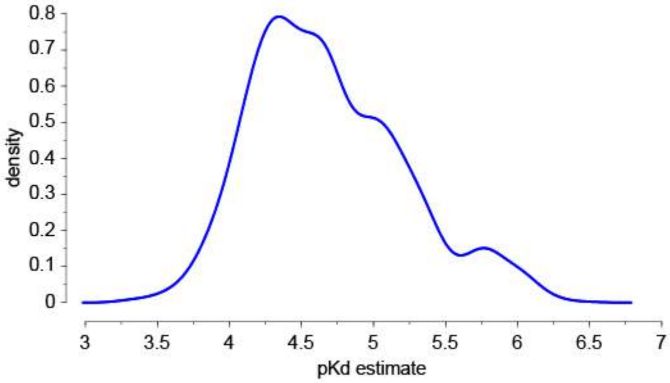

Figure S11. Density of dsrm-RNA affinity estimates has a heavy right tail. We plot the kernel density function of pKd estimates over all nodes on the dsrm phylogeny (Fig. 4, main text), calculated across all structural models and using Gaussian kernel smoothing (see Methods for details).

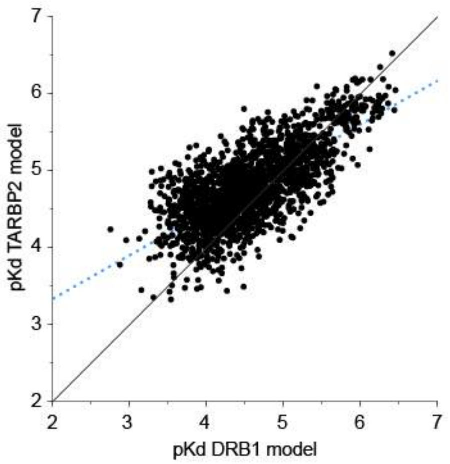

Figure S12. Affinity predictions from TARBP2 and DRB1 models are correlated. We plot the average protein-RNA pKd predicted for each node on the dsrm phylogeny (Fig. 4, main text), estimated over 5 replicate structural models using either the TARBP2 (Y-axis) or DRB1 (X-axis) structure as a template. Blue line indicates best-fit linear regression.

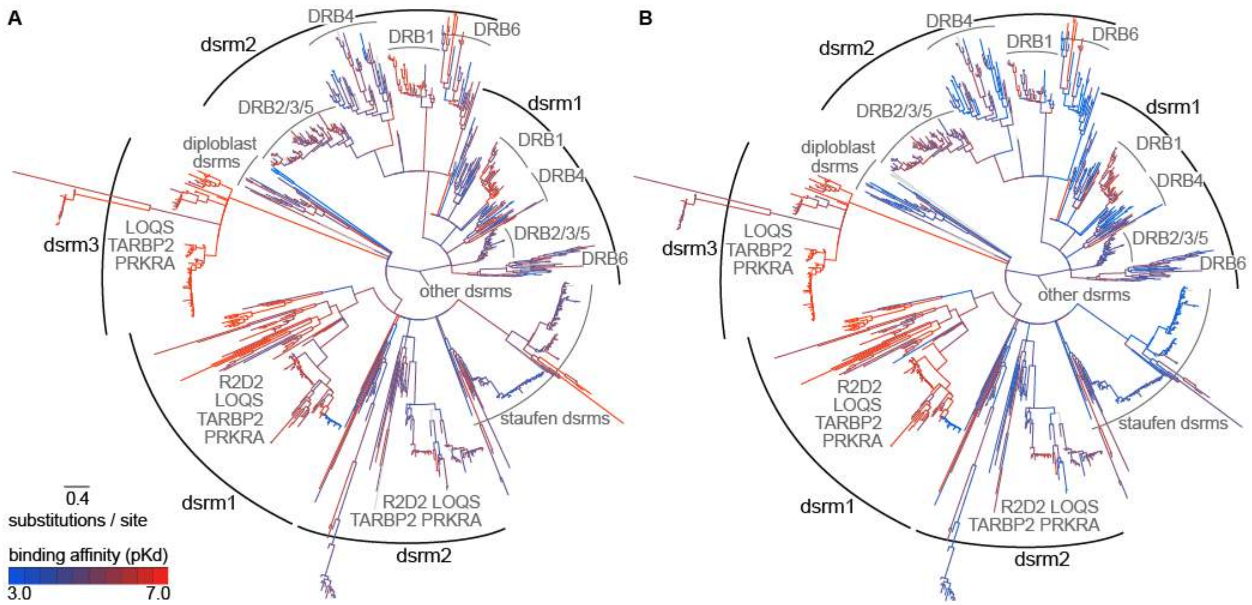

Figure S13. Mean affinity predictions across the dsrm phylogeny are similar using TARBP2 or DRB1 structural models. We reconstructed the maximum-likelihood domain phylogeny of dsrm functional domains from animal and plant DRB genes and plot a consensus tree (see Figs. 2,4, main text). Branch lengths are scaled to substitutions/site. Maximum-likelihood ancestral sequences were reconstructed at each node on the phylogeny, and we inferred the structure of all ancestral and extant dsrm protein sequences bound to dsRNA by homology modeling and molecular dynamics, using either TARBP2 (**A**) or DRB1 (**B**) structures as templates (see Methods). Inferred dsrm-RNA complexes were used to predict RNA binding affinities. We plot the average dsrm-RNA binding affinity (pKd) across multiple replicate models of each ancestral and extant sequence on the phylogeny, with red indicating high-affinity and blue indicating low-affinity.

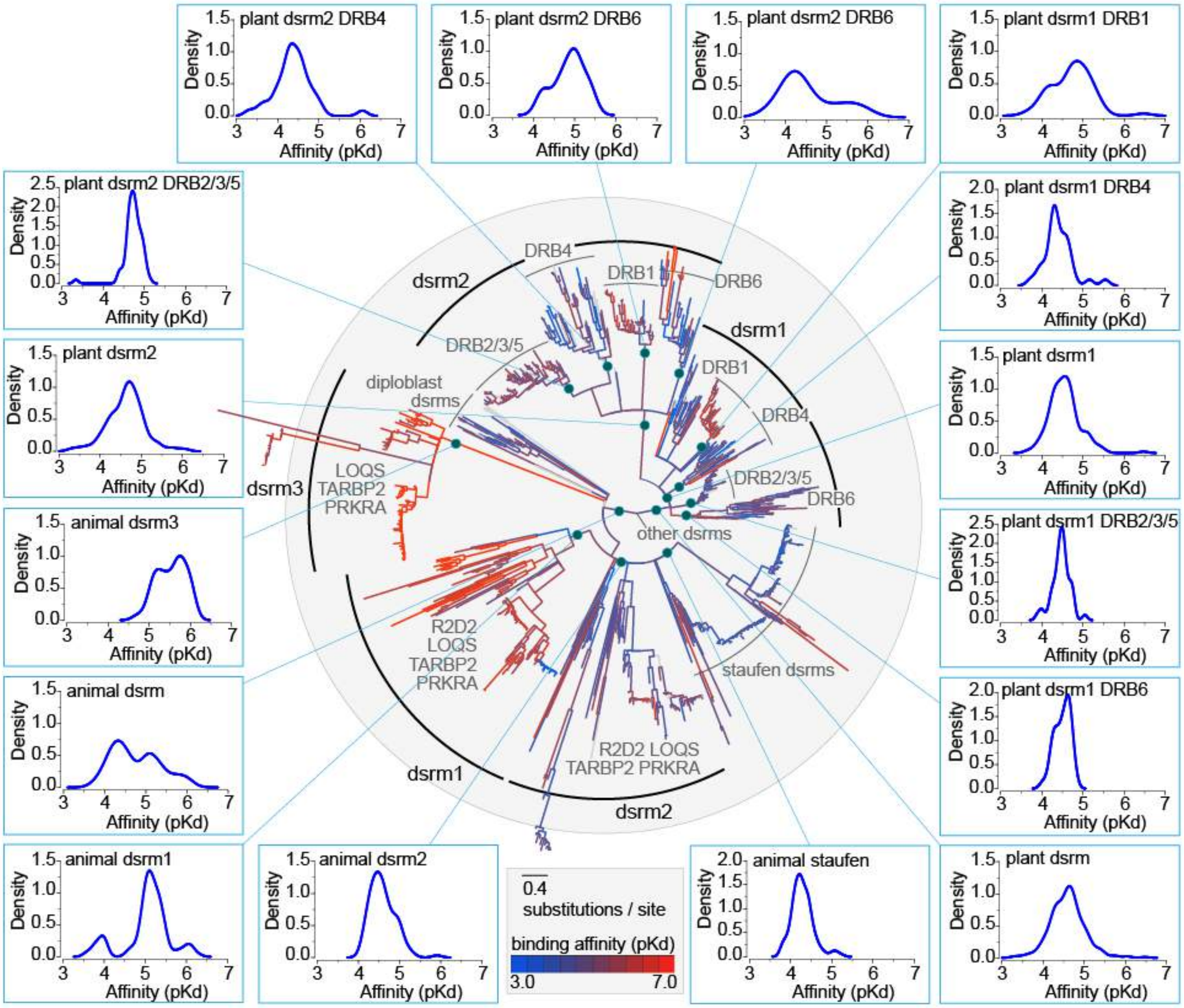

Figure S14. Predicted dsrm-RNA affinities differ across phylogenetic groups. For key nodes on the DRB dsrm phylogeny, we plot the gaussian kernel density of RNA affinities (predicted pKd values) for all ancestral and extant sequences descended from that node. Higher pKd values indicate greater affinity for dsRNA. Branches are scaled to the expected number of protein substitutions/site and colored by the mean pKd of the descendent node of the branch, estimated over replicate structural models (see Methods).

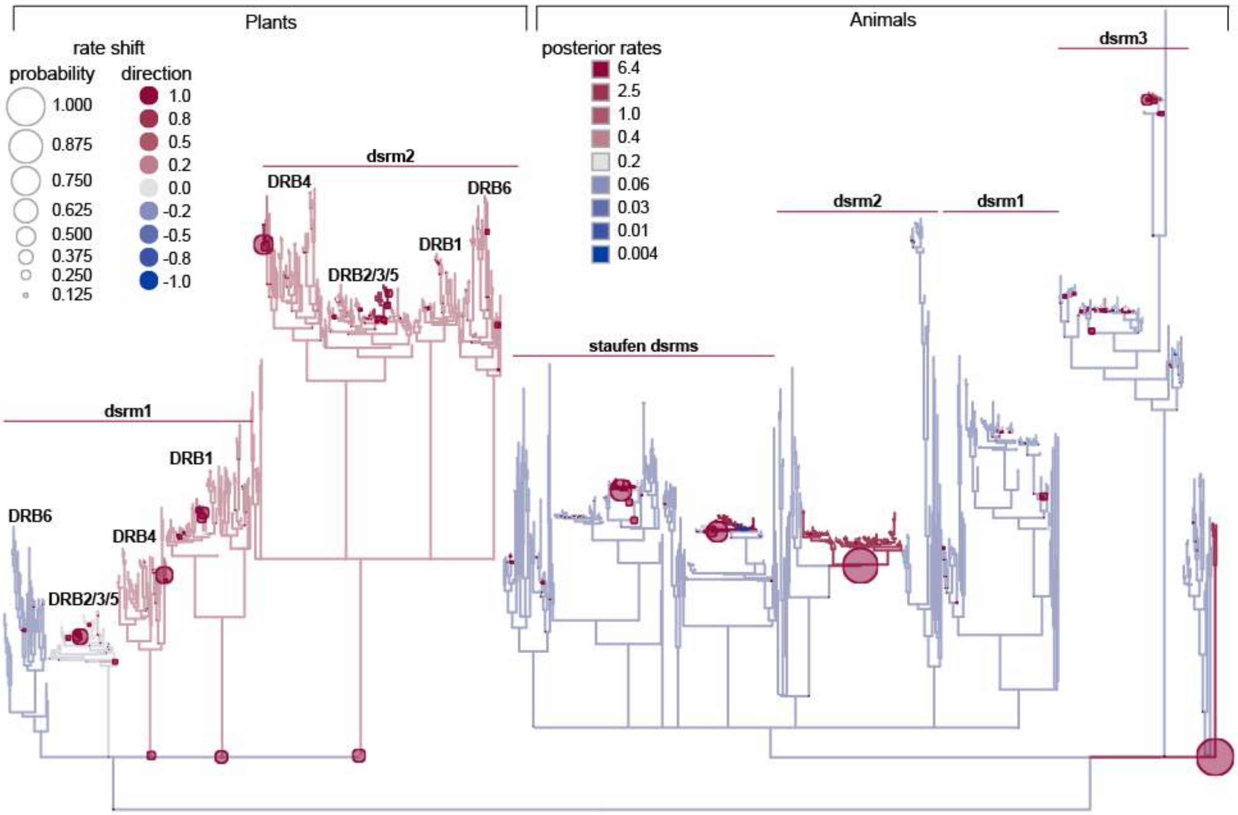

Figure S15. The rate of dsrm-RNA affinity evolution is higher in plants than in animals and exhibits a number of discrete shifts across the dsrm phylogeny. We inferred the evolution of the rate at which dsrm-RNA affinity changes using a Brownian motion 'break point' model of affinity evolution fit to predicted dsrm-RNA affinities using only extant sequences (see Methods). Branches are scaled to the inferred number of protein substitutions/site and colored by the posterior rate multiplier, averaged over 4 independent MCMC chains. Red branches indicate faster evolution of dsrm-RNA affinity, with blue branches indicating slower evolution of affinity. Circles on nodes indicate inferred increases (red) or decreases (blue) in the rate multiplier, with the size of the circle indicating the posterior probability of a discrete shift at the specified node. Outgroup branches have been removed. Major taxonomic and gene family lineages are indicated. The results presented here are similar to those in Figure 5 (main text), except this model was fit using only affinity predictions from extant sequences, whereas the model presented in Figure 5 (main text) was fit to affinity data from both extant and ancestral-reconstructed sequences.

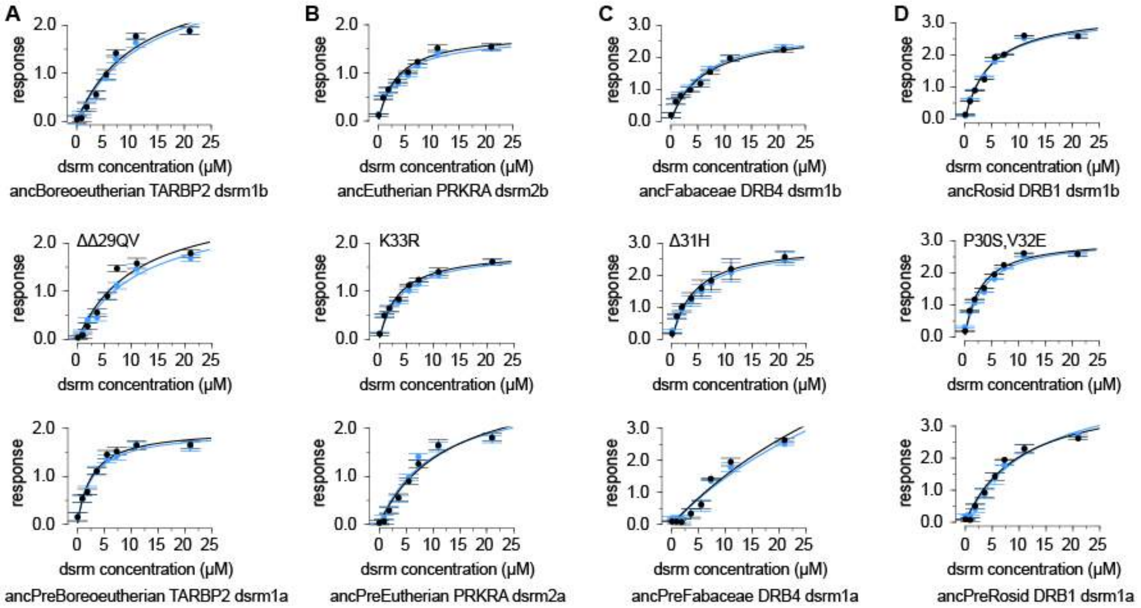

Figure S16. Observed shifts in animal and plant dsrm-RNA affinities are explained by substitutions in the β1-β2 loop and the α2 region. We measured steady-state (black) and initial (blue) binding of each ancestral protein indicated in Figure 6 (main text) to double-stranded RNA using a label-free kinetics assay across increasing dsrm concentrations (see Methods). Historical substitutions at key RNA-contacting residues were introduced by site-directed mutagenesis. Lines indicate best-fit single-site binding curves (r^2>0.90), and bars indicate standard errors over three replicates. Panels (A-D) correspond to those in Figure 6 (main text).

Supplementary References

Enright AJ, Van Dongen S, Ouzounis CA. 2002. An efficient algorithm for large-scale detection of protein families. Nucleic Acids Res 30:1575-1584.

Sawyer S. 1989. Statistical tests for detecting gene conversion. Mol Biol Evol 6:526-538.

Wilson RC, Tambe A, Kidwell MA, Noland CL, Schneider CP, Doudna JA. 2015. Dicer-TRBP complex formation ensures accurate mammalian microRNA biogenesis. Mol Cell 57:397-407.

Yang SW, Chen HY, Yang J, Machida S, Chua NH, Yuan YA. 2010. Structure of Arabidopsis HYPONASTIC LEAVES1 and its molecular implications for miRNA processing. Structure 18:594-605.
